# Sporadic Parkinson’s disease derived neuronal cells show disease-specific mRNA and small RNA signatures with abundant deregulation of piRNAs

**DOI:** 10.1186/s40478-018-0561-x

**Published:** 2018-07-10

**Authors:** Markus Schulze, Annika Sommer, Sonja Plötz, Michaela Farrell, Beate Winner, Janina Grosch, Jürgen Winkler, Markus J. Riemenschneider

**Affiliations:** 1 Department of Neuropathology, Regensburg University Hospital, Franz-Josef-Strauss-Allee 11, 93053 Regensburg, Germany; 2 Department of Stem Cell Biology, Friedrich-Alexander University (FAU) Erlangen-Nürnberg, Erlangen, Germany; 3 Department of Molecular Neurology, FAU Erlangen-Nürnberg, Erlangen, Germany; 4 Present address: Division of Molecular Genetics, German Cancer Research Center (DKFZ), Im Neuenheimer Feld 580, 69120 Heidelberg, Germany

**Keywords:** Methylation, PD, Induced pluripotent stem cells, iPSC, SINE, LINE

## Abstract

Differentiated neurons established via iPSCs from patients that suffer from familial Parkinson’s disease (PD) have allowed insights into the mechanisms of neurodegeneration. In the larger cohort of patients with sporadic PD, iPSC based information on disease specific cellular phenotypes is rare. We asked whether differences may be present on genomic and epigenomic levels and performed a comprehensive transcriptomic and epigenomic analysis of fibroblasts, iPSCs and differentiated neuronal cells of sporadic PD-patients and controls. We found that on mRNA level, although fibroblasts and iPSCs are largely indistinguishable, differentiated neuronal cells of sporadic PD patients show significant alterations enriched in pathways known to be involved in disease aetiology, like the CREB-pathway and the pathway regulating PGC1α. Moreover, miRNAs and piRNAs/piRNA-like molecules are largely differentially regulated in cells and post-mortem tissue samples between control- and PD-patients. The most striking differences can be found in piRNAs/piRNA-like molecules, with SINE- and LINE-derived piRNAs highly downregulated in a disease specific manner. We conclude that neuronal cells derived from sporadic PD-patients help to elucidate novel disease mechanisms and provide relevant insight into the epigenetic landscape of sporadic Parkinson’s disease as particularly regulated by small RNAs.

**Electronic supplementary material:**

The online version of this article (10.1186/s40478-018-0561-x) contains supplementary material, which is available to authorized users.

## Background

Coding mutations like the well-known A53T α-synuclein and G2019S LRRK2 mutations cause familial Parkinson’s disease (PD) [38]. Dopaminergic neurons carrying these mutations obtained via an induced pluripotent stem cell (iPSC) intermediate have been shown to recapitulate hallmarks of the neurodegenerative process in PD like increased susceptibility to oxidative [42] and nitrosative stress [53].

Much less is known about the possibility to model sporadic PD with iPSC based models. In sporadic PD that constitutes about 90% of cases and where none of the well-known genes causing familial Parkinsonism is mutated, no single variant in the whole coding sequence of the human genome was found to be associated with PD in a recent study [55]. In contrast, alterations in non-coding regions, which are enriched in PD-related genes, are well established risk factors for PD [12]. In addition, alterations of the epigenome in sporadic Parkinson’s disease have been reported in brain tissue on methylation [25] and small RNA level [26] and epigenetic alterations are also present in the patients’ peripheral blood [24]. Although iPSC reprogramming has been reported to remove all marks associated with cellular ageing [39], disease-specific alterations may survive reprogramming and then can impact iPSC function [16], even allowing for imprinting disorders to be modelled with iPSCs [7]. Importantly, both small RNAs [62] as well as methylation [43] can contribute to somatic memory.

Differential expression of disease associated small RNAs in cultured, differentiated neurons would allow to recapitulate and to study epigenome-mediated pathological alterations. Mature miRNAs, which repress target gene functions both by regulating target mRNA levels as well as by repression of target mRNA translation, have already been implicated in the pathogenesis of Parkinson’s disease, e.g. in animal models [47]. PIWI interacting RNAs, a class of small regulatory RNAs first described in the male germline [13], are less well studied in neuronal cells but have been implicated in retrotransposon silencing in the brain [41] as well as regulation of epigenetic modifications [49]. Importantly, piRNAs were recently described to be differentially regulated in Alzheimer’s disease brain tissue [48, 52] connecting them to the process of neurodegeneration.

Most phenotypes observed in neurons differentiated from cells carrying a mutation that causes familial PD can only be observed after the cells have been stressed, implying that the alterations in the coding sequence lead to a stress-susceptible, primed state in the neurons. More specifically, increased cell death in neurons carrying the A53T mutation in α-synuclein is observed after treatment with mitochondrial toxins [53] and in neurons carrying the G2019S LRRK2 mutation after H_2_O_2_ application [42].

Under certain circumstances, dopaminergic neurons derived from sporadic Parkinson’s disease patients have been reported to show phenotypes similar to genetic cases including cellular abnormalities [54], altered methylation patterns [12] as well as abnormalities in protein turnover [18]. Importantly, the epigenomic alterations observed were present in pathways important for physiological neurodevelopment [12] supporting the hypothesis that these pathways are altered in PD [29] as has been reported for Alzheimer’s disease [14]. The alterations in protein turnover were already present in fibroblasts [18] and a large subset of fibroblasts derived from sporadic PD cases showed increased sensitivity to valinomycin treatment [58]. As fibroblasts are frequently used for reprogramming to iPSCs followed by differentiation into neuronal cells, it is possible that an epigenetic signature survives reprogramming that potentially impacts the function of transplanted cells. More importantly, this signature -if present- might provide unique insights into the disease aetiology of sporadic PD. Therefore, the Bavarian Research Network Induced Pluripotent Stem Cells (ForIPS) was initiated to test the hypothesis, which -if any- phenotypes can be observed in cells derived from sporadic PD-patients.

Based on these premises, we examined a large region specific cohort of matched fibroblasts, iPSCs and differentiated neuronal cells from healthy individuals and Parkinson’s disease patients and applied rigorous quality control standards. Previous to our analyses, we confirmed the PD-patients to be negative for all known monogenic forms of the disease. We aimed to determine in a global and unbiased approach if there were any epigenetic marks that distinguish cells from healthy and diseased individuals. Indeed, we found that cells derived from sporadic Parkinson’s disease patients show a unique small RNA signature and that pathways that are known to be altered in diseased tissue as well as genetic cases, like the pathway regulating PGC1α, are deregulated.

## Methods

### Cell culture, iPSC generation and differentiation to midbrain neurons

Induced pluripotent stem cells (iPSC) were generated from eight sporadic PD patients and six healthy controls by retroviral transduction of the four transcription factors (Oct 3/4, c-Myc, Sox2 and Klf4) as previously described [15]. The Institutional Review Board approval (Nr. 4120: Generierung von humanen neuronalen Modellen bei neurodegenerativen Erkrankungen) and informed consent forms are on file at the movement disorder clinic at the Department of Molecular Neurology, Universitätsklinikum Erlangen (Erlangen, Germany). All the hiPSC lines were cultured in mTeSR (Stemcell Technologies) or MACSbrew (Miltenyi) on Geltrex (Gibco® Thermo Fisher Scientific) and splitted every five to seven days using Collagenase IV or Gentle Cell Dissociation Reagent (Stemcell Technologies). Pluripotency and a stable karyotype were tested by flow cytometry and G-banding chromosomal analysis, respectively. One to two hiPSC clones per individual (Additional file 1: Table S1) were differentiated into small molecule neural precursor cells (smNPCs) following the protocol published by others [50] with some adaption as described in [59]. The smNPCs were further differentiated to midbrain neurons within three weeks of maturation [50, 59]. Briefly, 70% confluent iPSC were detached by collagenase IV (Gibco® Thermo Fisher Scientific) treatment for 20 min at 37 °C, 5% CO_2_. Cell colonies were cultured as free-floating aggregates in human embryonic stem cells (hESC) medium (80% KO-DMEM, 20% KO serum replacement, 1% non-essential amino acids, 1% Penicillin/Streptavidin (all from Thermo Fisher Scientific), 1 mM ß-Mercaptoethanol (Sigma-Aldrich) supplemented with the small molecules 1 μM LDN (Stemgent), 10 μM SB, 3 μM Chir, and 0.5 μM Purmorphoamine (PMA, all from Tocris) on ultra-low adhesion plates. After two days of incubation at 37 °C, 5% CO2, the cell colonies were centred and the medium was changed to N2B27 medium (50% DMEM/F12, 50% Neurobasal Medium, 1:200 N2, 1:100 B27 (all from Thermo Fisher Scientific) supplemented with the same small molecules. On day four, the medium was changed to smNPC medium (N2B27 medium supplemented with 3 uM Chir, 0.5 uM PMA and 150 uM Ascorbic acid (AA; Sigma-Aldrich). After a total of six days of suspension culture, cell colonies were replaced on geltrex-coated (Gibco® Thermo Fisher Scientific) 12-well plates in smNPC medium supplemented with Rho kinase inhibitor Y27532 (RI, Axxora) for 24 h. Medium was changed every other day and cells were passaged once a week by accutase treatment. After at least five passages, smNPCs were differentiated into MN. Therefore, two days after passaging, the medium was exchanged to N2B27 medium supplemented with 100 ng/ml FGF8 (Peprotech), 1 μM PMA and 200 μM AA. On day 10 of differentiation, medium was supplemented with 100 ng/ml FGF8, 10 ng/ml GDNF (Peprotech), 10 ng/ml TGFb (eBioscience), 200 uM AA, and 500 μM Dibutyryl-cAMP (dbcAMP; Sigma-Aldrich. On the next day, cells were passaged at ratios of 1:2–1:3 as single cells after accutase treatment (Sigma-Aldrich), plated onto geltrex-coated four-well chamber slides (Ibidi) or 12-well plates and further cultured for at least two weeks in maturation medium (N2B27 medium plus 100 ng/ml FGF8, 10 ng/ml GDNF (Peprotech), 10 ng/ml TGFb (eBioscience), 200 uM AA, and 500 μM Dibutyryl-cAMP (dbcAMP; Sigma-Aldrich) with two times media change per week.

### Poly-a RNA library preparation

Libraries for next-generation sequencing were prepared from 1 μg total RNA with the TrueSeq RNA library preparation kit v2 according to the manufacturer’s instructions (Illumina, San Diego, CA, USA). Briefly, poly-A RNA was purified from the total RNA preparation with magnetic oligo-dT beads. The RNA-bead mixture was incubated at 65 °C for five minutes to denature the RNA. Then, the mixture was incubated for five minutes at RT to allow the RNA to bind to the beads. Afterwards, the beads were washed with bead washing buffer and resuspended in elution buffer for two minutes at 80 °C. Bead binding buffer was added to allow rebinding of the eluted RNA. The beads were washed with bead washing buffer and resuspended in elute, prime, fragment mix. The RNA was eluted at 94 °C for eight minutes, and first strand cDNA synthesis was performed with SuperScript II (Thermo Fisher Scientific, Waltham, MA, USA) in first strand mix supplied by Illumina. Second strand synthesis was performed with the second strand mix for one hour at 16 °C and the resulting double stranded cDNA was purified using AMPure XP beads (Beckman Coulter, Brea, CA, USA). Then, end repair was performed for 30 min at 30 °C with the provided end repair mix. The end repaired DNA was again cleaned up with AMPure XP beads. A-tailing was performed with the A-tailing mix at 37 °C for 30 min followed by 70 °C for five minutes. Indexed adapters were ligated to the end-repaired A-tailed cDNA at 30 °C for ten minutes. Next, stop ligation buffer was added and the libraries were cleaned up with AMPure XP beads. PCR amplification was performed with the provided PCR reagents and the following cycling conditions: Denaturation at 98 °C for 30 s and then 15 cycles of1) 98 °C for 10 s, 2)60 °C for 30 s and 3)72 °C for 30 s. Afterwards, a final extension at 72 °C for five minutes was performed and the amplified libraries were purified again with AMPure XP beads. Finally, quality control was performed with a Bioanalyser® (Agilent).

### RNA library preparation for tissue samples

For all tissue samples (RIN usually < 8), we used the TrueSeq RNA Access Kit (Illumina) to prepare libraries from 100 ng total RNA, which is suitable for degraded RNA. First, first strand cDNA was synthetized with the Elute, Prime, Fragment High Mix and super script II in First Strand Master Mix (25 °C for 10 min, 42 °C for 15 min and 70 °C for 15 min). Then, 20 μl Second Strand Marking Master Mix were added and the second strand was synthetized for 1 h at 16 °C. The cDNA was cleaned up with AMPure XP beads and A-tailing was performed with A-tailing mix (37 °C for 30 min followed by 5 min at 70 °C). Following this, adapter ligation was performed with Ligation Mix for 10 min at 30 °C after which the reaction was stopped with Stop Ligation Buffer. The libraries were cleaned up with AMPure XP beads and amplified with PCR Master Mix and PCR Primer Cocktail (98 °C for 30 s, 15 cycles of 98 °C for 10 s, 60 °C for 30 s, 72 °C for 30 s and 72 °C for 5 min). Following a further clean up with AMPure XP beads, the libraries were quantitated with a Bio-Analyser and 4 libraries were pooled at equal concentrations (200 ng each) for exome capture, which was repeated twice. For exome capture, Coding Exome Oligos were added to the pooled libraries together with Capture Target Buffer 3 and incubated for 95 °C for 10 min and 18 cycles of one minute incubations, starting at 94 °C, then decreasing 2 °C per cycle with a final incubation for 58 °C for 90 min. Immediately afterwards, the hybridized probes were captured with streptavidin magnetic beads for 25 min at RT and washed twice with Enrichment Wash Solution (incubation at 50 °C for 20 min). Finally an Elution Premix was prepared with Enrichment Elution Buffer 1 and NaOH. The streptavidin beads were resuspended in this Elution Premix, incubated for two minutes at RT, the supernatant was separated from the beads on a magnetic stand and Elute Target Buffer 2 was added to the supernatant. After the second exome capture, the libraries were cleaned up with AMPure XP beads. Finally, a second enrichment was performed with the Enhanced PCR Mix (98 °C for 30 s followed by 10 cycles of 98 °C for 10 s, 60 °C for 30 s and 72 °C for 30 s, with a final extension at 72 °C for 5 min) and the amplified libraries were purified again with AMPure XP beads. Finally, quality control was performed with a Bioanalyser® (Agilent).

### Reduced representation bisulfite sequencing (RRBS) library preparation

Reduced representation bisulfite sequencing allows for highly accurate and efficient analysis of methylation patterns at single base pair resolution with a focus on CpG islands [21]. Digestion was performed with 2.5 μg gDNA in buffer 4 (NEB) and 400 units MSPI O/N at 37 °C. Afterwards, the DNA was purified from this reaction with the PCR purification Kit (Qiagen) according to the manufacturer’s instructions. Briefly, 5 volumes buffer PB were added to the MSPI digest, applied to a spin column provided in the kit and centrifugation was performed for 1 min at 9500 *× g*. Then, the column was washed with 750 μl buffer PE, dried by centrifugation for 1 min at 9500 *× g* and the DNA was eluted with 30 μl buffer EB. Library preparation was then performed with the NEXTflex™ Bisulfite Library Prep Kit (BIOO Scientific) according to the manufacturer’s instructions with some modifications. Briefly, end repair was performed with 500 ng digested, purified DNA in end repair buffer mix and end repair enzyme mix in a total volume of 50 μl. The reaction was incubated at 22 °C for 30 min and then cleaned up with the MinElute® PCR Cleanup Kit. Then, 16.5 μl of the eluate were mixed with 4.5 μl of adenylation mix and the reaction was incubated for 30 min at 37 °C. Afterwards, 31.5 μl ligation mix and 2.5 μl of individual adapters (diluted 1:2) were added, and adapter ligation was performed for 15′ at 22 °C. Afterwards, the DNA was cleaned with AMPure XP beads and size selection for fragments from 175 to 400 bp was performed with a gel purification step. The libraries were separated on a 2% low melt agarose gel (Sigma-Aldrich), the cut out gel fragments were dissolved for 10 min at RT in DNA binding buffer and 150 μl ethanol were added. Then, the solution was applied to a clean-up spin column and centrifuged at 18500 *xg* until the complete volume was processed. Afterwards, the column was washed twice with DNA wash buffer, dried by centrifugation and the DNA was eluted with column elution buffer. Then, bisulfite conversion of the DNA was performed with the EZ Methylation Gold Kit (Zymo Research) according to the manufacturer’s instructions. Briefly, 130 μl conversion reagent were added to 20 μl purified DNA. The reaction was incubated for 10 min at 98 °C and for 2.5 h at 64 °C. Then, the samples were loaded on spin columns containing 600 μl M-Binding buffer and mixed by inverting. The DNA was bound to the column by centrifugation for 30 s at 18620 *x g*. Then, the column was washed with 100 μl wash buffer, and 200 μl desulphonation buffer were added. The desulphonation buffer was incubated for 17 min at RT, and then removed by centrifugation. The column was washed twice with 200 μl wash buffer, and dried by centrifugation for 10 s. Finally, 17 μl elution buffer were added to the column, incubated for 1 min and the DNA was eluted by centrifugation. Afterwards, PCR amplification of the bisulfite converted libraries was performed with PfuCx Hot Start (Agilent). 15 μl DNA were amplified with the NEXTflex™ Primer Mix. Cycling conditions were: Initial denaturation for five minutes at 95 °C. Then 18 cycles of 95 °C for 30 s, 65 °C for 30 s and 72 °C for 45 s. Afterwards a final extension at 72 °C for 7 min. The libraries were purified again using AMPure XP beads (Beckmann Coulter). Finally, quality control with a Bioanalyser® (Agilent) was performed.

### Small RNA library preparation

Small RNA libraries were prepared from 1 μg total RNA containing small RNAs with the TrueSeq Small RNA kit (Illumina) according to the manufacturer’s instructions. Briefly, a 3’adapter was added to the RNA, denaturation was performed for two minutes at 70 °C and afterwards the mixture was put on ice immediately. Then, the adapter was ligated with a T4 RNA Ligase2 deletion mutant (epicentre) for 1 h at 28 °C. Then the reaction was stopped with stop solution for 15 min at 28 °C, the previously denatured 5′ adapter was added together with ATP and T4 DNA Ligase and ligated to the RNA for 1 h at 28 °C. After that, cDNA synthesis was performed with super script II and Illumina supplied dNTPs for 1 h at 50 °C. Afterwards, the cDNA was amplified and indexed with the primers and PCR mix supplied in the kit (eleven cycles of denaturation at 98 °C for 10 s, annealing 60 °C for 30 s and extension at 72 °C for 15 s with a final extension at 72 for 10 min) and size selection was performed on 5% TBE acrylamide gels (Bio-Rad, Munich, Germany). Here, the region marked by Illumina’s custom ladder between 145 bp and 160 bp were cut out and pooled for sequencing. The gel was homogenized by centrifugation at 20000 x *g* through a gel breaker tube (Bio-Cat) and 300 μl ultrapure H_2_O were added to elute the DNA O/N in a DNA LoBind tube (Eppendorf). The following day, the gel debris was separated from the water by centrifugation for 10 s at 600 *xg* through a 5 μm filter tube (Bio-Cat). Then, 2 μl glycogen (CALBIOCHEM), 30 μl sodium acetate (Thermo-Fisher Scientific), 2 μl 0.1× pellet paint and 975 μl prechilled 100% ethanol were added and the mixture was incubated for 20 min at − 80 °C. Then, the library was pelleted by centrifugation for 20 min at 4 °C and 20,000 xg, washed with 70% ethanol and recentrifuged for 5 min. Finally, the ethanol was removed, the pellet dried for five minutes at 37 °C and resuspended in 15 μl ultrapure water.

### Next-generation sequencing

Illumina deep sequencing as well as quantification of small RNA content were performed at a genomics core facility: Center of Excellence for Fluorescent Bioanalytics (KFB, University of Regensburg, Germany). For deep sequencing, all libraries were quantified using the KAPA SYBR FAST ABI Prism Library Quantification Kit (Kapa Biosystems, Woburn, MA, USA). Equimolar amounts of each library were used for cluster generation on the cBot with the TruSeq SR Cluster Kit v3 (Illumina, San Diego, CA, USA). The sequencing run was performed on a HiSeq 1000 instrument (Illumina, San Diego, CA, USA) using the indexed, 50 cycles single read (SR) protocol and the TruSeq SBS v3 Kit (Illumina, San Diego, CA, USA). Image analysis and base calling resulted in .bcl files which were then converted into .fastq files by the CASAVA1.8.2 software.

### Data analysis for (small) RNA libraries

Analysis of poly-A RNA, RNA enriched with the coding exome oligos and small RNA data was performed using the Genomatix software (Genomatix, Munich, Germany). For poly-A RNA and RNA enriched with the coding exome oligos, the .fastq files were mapped to the human genome assembly GRCh38 (annotation based on ElDorado 6–2015) using the Genomatix Mining Station Mapper v3.7.6.3 allowing one mismatch. We sequenced all poly-A RNA libraries until at least 15 * 10^6^ and all exome capture libraries until at least 10 × 10^6^ unique hits per sample could be mapped. All unique hits were further processed using the Genomatix Genome Analyser v3.51106 which was used to create count tables and RPKM expression values for all samples. Reads were counted locus-based, i.e. for unions of exons of genes. All further analyses based on these count tables were performed with the free software R v3.1.1, Bioconductor v3.0 [20] and the package DESeq2 v1.6.3 [36]. Gene set enrichment analysis [53] was performed with GenePattern and the GSEA module v18 [53] with RPKM (reads per kilobase of exon model per million mapped reads) values and gene sets c2.Biocarta as well as c2.KEGG using standard settings. For small RNA analysis, we mapped .fastq files against a small RNA library based on GRCh38 (annotation based on ElDorado 6–2015) allowing one mismatch. Afterwards, count tables were created for each small RNA type (piRNA, mature miRNA, miRNA hairpin structures) with the small RNA workflow available in the Genomatix Genome Analyser. We sequenced all samples until at least 1 × 10^6^ counts for mature miRNAs were reached when reads were counted, with a mean of unique hits for mature miRNAs of 3,037,413 ± 2088,481and for piRNA 9,754,231 ± 4,096,709. All further single locus based analyses were performed as described for poly-A RNA. For further analysis of piRNAs, we matched the RNAdb2.0 identifiers [45], on which Genomatix is based, to the pIRbase [66], which provides information on the genomic localization, sequence as well as the elements from which the respective piRNAs are derived. Parts of the poly-A RNA-Seq dataset were generated in a collaborative project and three poly-A RNA-Seq runs from neurons are also contained/analysed in [59].

### RRBS analysis

For RRBS, .fastq files were trimmed with TRIM Galore v0.4.2 in the rrbs mode and mapped with BISMARK v0.16.3 [28] to the human genome GRCh38.84 as obtained from www.ensembl.org. Then, the methylation values were extracted with the BISMARK methylation extractor again removing 2 bp at the five prime end of the reads to remove the methylation bias in untrimmed reads stemming from the end repair procedure. We sequenced the libraries until at least 1 × 10^6^ alignments with a unique best hit were found by BISMARK. All further analyses were performed based on extracted coverage files with RnBeads v1.2.0 [4].

### Data availability

All normalized NGS data were deposited in GEO (URL: https://www.ncbi.nlm.nih.gov/geo) under the super series accession GSE110720. Coding exome RNA-Seq data is deposited under accession GSE110716, Poly-A RNA-Seq data is deposited under accession GSE110717, RRBS data is deposited under accession GSE110718 and small RNA-Seq data under accession GSE110719.

### Quality control and inclusion criteria

Due to historical reasons, the mRNA data for quality control analyses of iPSCs were mapped to the human genome GRCh37 without mismatch. We included all samples that we analysed, as long as they met the following criteria: iPSCs had to show a marker expression of the pluripotency markers DNMT3B, SOX2, NANOG, OCT4 and REX1 within the range of a previously analysed high quality ESC cohort and to fall at least in the safety margin of a previously established database driven PluriTest adaption [57]. Furthermore, viral transgene silencing had to be comparable to a published cohort for which NGS raw data was freely available [1]. We analysed viral transgene silencing both by comparison of RPKM values of established markers of viral transgene silencing [27] as well as by direct mapping of the plasmids used for reprogramming counting multiple and unique hits when mapping against the vector sequence (excluding the coding regions of the pluripotency factors) normalized to the number of unique hits of the respective sequencing run when mapped to the genome. The upper-limit cut-offs shown in the supplementary material were calculated as described in [57]. SNPs were called within the Genomatix Genome analyser with a workflow based on samtools, with at least 4 x coverage per SNP and exclusion of indels. We excluded five iPSC lines based on these criteria.

### Patient samples, tissue samples and embryonic stem cells

The Institutional Review Board approval (Nr. 4120: Generierung von humanen neuronalen Modellen bei neurodegenerativen Erkrankungen) and informed consent forms are on file at the movement disorder clinic at the Department of Molecular Neurology, Universitätsklinikum Erlangen (Erlangen, Germany). All procedures involving patient samples (tissues or cells) were approved by the local institutional review board (Ethikkommission Regensburg), approval 14–101-0216. The experiments involving embryonic stem cells were approved by the Central Ethics Committee for Stem Cell Research in Germany according to StZG (AZ: 3.04.02/0121). Tissue samples were obtained from the Netherlands brain bank as fresh frozen tissue. iPSCs were generated from skin biopsies of PD- and control-patients by the ForIPS core project as described elsewhere [15].

### Immunohistochemistry

The presence of Lewy bodies in the substantia nigra and more importantly in the cingulum was verified with stained sections from the Netherlands brain bank (NBB). For those cases were no staining was available, we obtained paraffin sections from the NBB and performed a staining with an antibody directed against aggregated α-synuclein (anti-human α-synuclein 5G4, mouse monoclonal, analytikjena, Jena, Germany). After deparaffinization, antigen retrieval was performed by cooking in citrate buffer for 20 min and DAB staining was performed with the Envision Dual Link System-HRP DAB+ Kit (Agilent, Santa Clara, CA, USA) according to the manufacturer’s instructions. Briefly, the sections were blocked with Dual Endogenous Enzyme Block for 10 min and rinsed with PBS. Then, primary antibody was applied (diluted 1:500 in PBS + 1% BSA) for 40 min and again the slides were washed with PBS. Afterwards, labelled polymer was added for 30 min. After a further washing step, substrate was added for five minutes. Afterwards, the slides were washed in running tap water, counterstained in hemalaun (Dako/Agilent) and again rinsed in running tap water. Finally, the slides were dehydrated in increasing ethanol concentrations and xylol and mounted in entellan mounting medium (Merck).

### Methyl-cytosine staining

For analysis of methyl-cytosine content in tissue, we cut frozen tissue sections from the cingulate gyrus material. The DAB staining was performed with the Envision Dual Link System-HRP DAB+ Kit (Agilent, Santa Clara, CA, USA) according to the manufacturer’s instructions with some modifications. After cutting and thawing, the sections were first fixed with 4% PFA for 15 min, which was necessary as we retrieved unfixed material. Then the sections were washed three times for five minutes in TBS. Afterwards, the slides were incubated for 30 min in 2 N HCl for antigen retrieval. The slides were washed twice with PBS and blocking was performed with the Dual Endogenous Enzyme Block reagent for 10 min at RT. After further washing in TBS, anti-methyl-cytosine (Epigentek, mouse monoclonal, clone 33D3) antibody was added in 1:400 dilution and incubated O/N at 4 °C in 1% normal goat serum (PAN Biotech, Aidenbach, Germany) in TBS + 0.3% TritonX. A mouse IgG (Thermo-Fisher) was used as negative control. The next day, the slides were washed three times, covered with labelled polymer and incubated for 30 min at RT. Then, after one washing with TBS, the sections were covered with chromogen for 10 min. Afterwards, the slides were washed in running tap water, counterstained in Mayer’s hemalaun and again rinsed in running tap water. Finally, the slides were dehydrated in increasing ethanol concentrations and xylol (Carl-Roth) and mounted in entellan mounting medium (Merck).

### cDNA synthesis, real-time PCR and semiquantitative PCR

cDNA synthesis was performed from 200 ng total RNA using random hexamer primers (Gene Link, Hawthorne, NY, USA) and the SuperScript™ II Reverse Transcriptase (Life Technologies) according to the manufacturer’s instructions. Real-time RT-PCR was performed with the SensiFAST™ SYBR Hi-Rox Kit (Bioline, London, UK) on the StepOnePlus™ cycler (Life Technologies). Relative expression values were calculated with the ΔΔC_T_ (analysis of relative gene expression) method [35] using *ARF-1* as the reference transcript. Primers used for *ARF-1* were 5´-GACCACGATCCTCTACAAGC (forward) and 5´-TCCCACACAGTGAAGCTGATG (reverse), for *TH* 5´-CCAAGACCAGACGTACCAGT (forward) and 5′- CGTGAGGCATAGCTCCTGAG (reverse). Primers for analysis of SYP, MAP2, TUBB3, EN1, NURR1, KCNJ6 and FOXA2 were described by others [34]. Primers for WNT3 5´-GGAGAGGGACCTGGTCTACTA (forward) and 5′- CTTGTGCCAAAGGAACCCGT (reverse) were and downloaded from the PrimerBank database [63]. Analysis of mtDNA content was performed with the real-time PCR conditions as described above and primers for mtDNA and nuclear DNA as described by others [51], as was analysis of mtDNA deletions [11]. Semiquantitative PCR was performed with the HotStarTaq polymerase (Qiagen) according to the manufacturer’s instructions and the equivalent of 125 ng cDNA per reaction. An annealing temperature of 55 °C and 35 cycles were used for amplification. Primers for semiquantitative PCR were designed with Primer3Plus [61]. Primer sequences for PIWIL2 were 5´-TTGGATTCGAAAATGGCTTC (forward) and 5´-AGCCAGGAAGCGGTTATTTT (reverse), PIWIL4 5´-CAAGGACGTGATGGTTGTTG (forward) and 5´-ACCGACAGTCTTGAGCTGGT (reverse). GAPDH was used as reference with the primers 5´-CCATCTTCCAGGAGCGAGAT (forward) and 5´-ATGATGTTCTGGAGAGCCCC (reverse).

### Statistics

All statistical analyses of NGS data were performed with the software packages described above and standard settings, unless otherwise indicated. A (small-) RNA locus was defined as differentially regulated when it was deregulated at a log2FC of ≥0.6 and an adjusted *p*-value of < 0.1. For analysis of methylation data a cytosine was accepted as differentially methylated when it passed the threshold Δ-meth ≥0.2 and an adjusted *p*-value of < 0.1. A gene set was accepted as differentially enriched at a *p*-value < 0.05 and a false discovery rate (FDR) < 0.25. Otherwise, all statistical tests used are provided in the figure legends and the results part together with the sample size. All bar graphs show the mean + standard deviation (SD) unless otherwise indicated.

## Results

### Patient recruitment, cell line establishment and quality control

We gained skin fibroblasts from six healthy control- and nine sporadic Parkinson’s disease (PD)-patients within the Bavarian Research Network Induced Pluripotent Stem Cells (ForIPS). All samples were derived from inner upper arm skin biopsy derived fibroblasts. Early passage fibroblast samples were reprogrammed to iPSCs using retroviral reprogramming Yamanaka factors. From all except one patient (PD7) iPSC lines were established, and finally a subset of these lines was further differentiated to midbrain neurons (MN) (Additional file 1: Table S1 for details on the patient cohort).

**Fig. 1:**
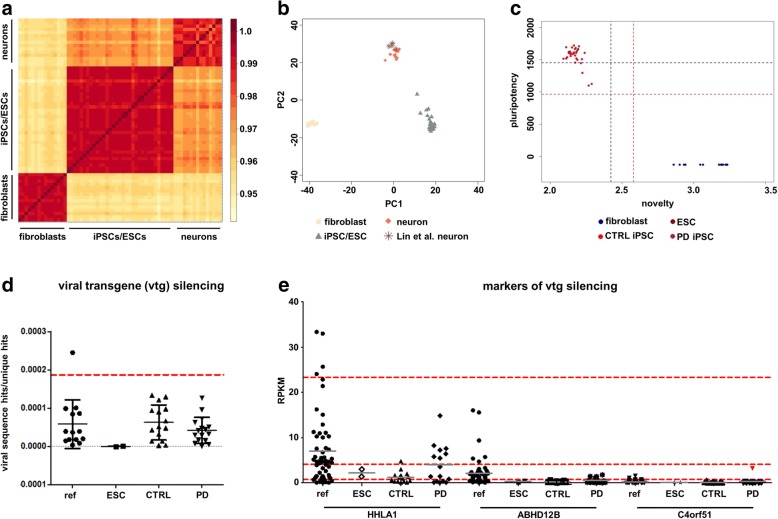
mRNA-based quality controls (**a**) Level plot of all correlation values of rlog normalized gene expression counts. Fibroblasts, iPSCs and neuronal cells show distinct gene expression patterns on a global scale. **b** Principal component analysis of fibroblasts, iPSCs/ESCs and neurons together with dopaminergic neurons from a literature cohort. The neurons generated cluster well with dopaminergic neurons available in databases [34]. **c** PluriTest [40] adaption [57] showing high similarity between embryonic stem cells and induced pluripotent stem cells in our cohort. **d** CTRL and PD iPSCs are comparable in their viral transgene silencing as well as in the range of a published iPSC cohort [1]. Human embryonic stem cells serve as a negative control. Only cells from databases were analysed that passed the pluripotency assessment. The red line depicts the statistical upper-limit cut-off calculated as described in [57]. **e** Expression analysis of published markers of insufficient viral transgene silencing. No cell line shows a consistent upregulation of markers of insufficient viral transgene silencing. The red line depicts the upper-limit cut-off calculated as described in [57]

Analysis of the transcriptome showed that fibroblasts, iPSCs and differentiated neuronal cells -as expected- had globally distinct gene expression patterns (Fig. 1a). Our neurons clustered well in a principal component analysis with dopaminergic neurons published by others [34] (Fig. 1b). We then validated that all iPSCs corresponded to high quality pluripotent cells according to our recent PluriTest adaption for NGS [57] (Fig. 1c). This means that all cell preparations mapped at least within a calculated statistical cut-off, with most preparations also meeting the requirements of an empirical cut-off of high quality pluripotent cells (for further information see [57]). We checked for the expression of pluripotency marker genes (in the range of high quality pluripotent cells, Additional file 2: Figure S1), ensured that cells showed viral transgene silencing (examined by direct mapping to the vector sequences used for reprogramming) and excluded that markers of insufficient viral transgene silencing were overexpressed [27] (Fig. 1d and e). In addition, we performed a SNP based paternity testing for every reprogrammed and differentiated line. All cell lines used for further analyses met these stringent quality criteria.

The neuronal cells showed robust induction of the dopaminergic neuron marker TH, and there was no significant difference between the control- and PD-group in the expression of TH, EN1, MAP2, FOXA2, KCNJ6, SYP2, TUBB3 and NURR1 (two-sided Mann-Whitney test, *p* > 0.05 for all comparisons, *n* = 8 CTRL and 7 PD derived MN neurons, Additional file 3: Figure S2). In addition the cells were not showing morphological differences or increased cell death without stressor [59] as has been described for genetic models of the disease [42, 53].

### Disease-specific phenotypes in differentiated neurons on mRNA level

**Fig. 2:**
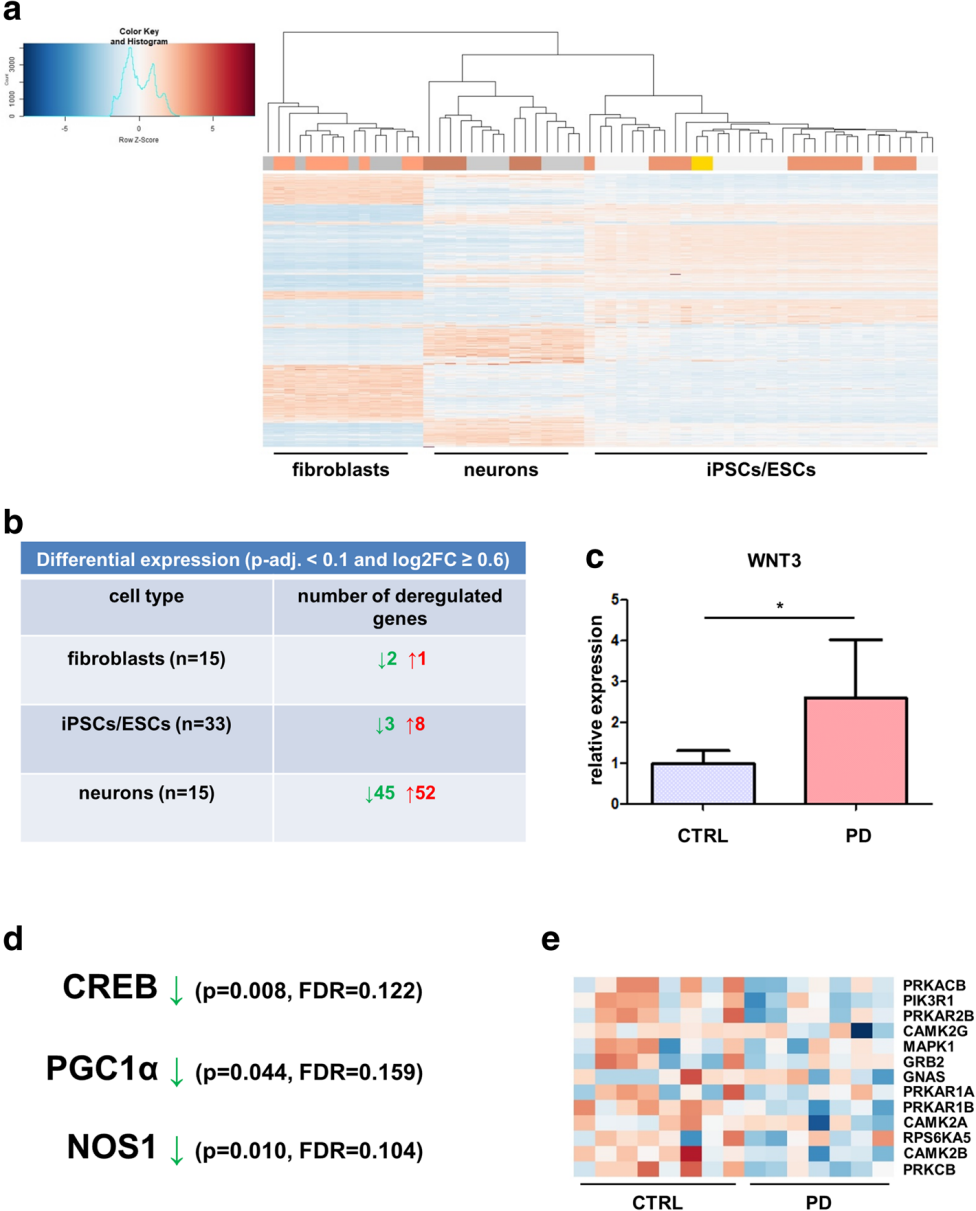
mRNA expression profiling of PD-patient derived cell lines (**a**) Heatmap and hierarchical clustering based on the TOP2000 variable genes on mRNA expression data. Hierarchical clustering separates the samples by cell type (fibroblast, iPSC/ESC or neuron). PD-patient derived cells (shades of salmon) are not separated from control-patient derived cells (shades of grey) and iPSCs are not separated from ESCs (gold). **b** Summary of differential expression analysis results as calculated by DESeq2 between control- and PD-patient derived cells. A significant number of deregulated genes is only present in neurons, but not in fibroblasts or iPSCs. **c** WNT3 is differentially expressed between control- and PD-patient derived cells as judged by real-time PCR (two-sided Mann-Whitney test, *p* < 0.05). Shown are means + SD. **d** The NOS1- and CREB-pathways as well as the pathway regulating PGC1α (as defined by c2.Biocarta) are significantly inactivated in PD-patient derived neuronal cells (*p* < 0.05 and FDR < 0.25 as calculated by GSEA). **e** Heatmap of CREB pathway genes that show a core enrichment in the GSEA analysis based on rlog normalised expression values of the midbrain neurons

A hierarchical clustering based on mRNA data (TOP2000 variable genes, rlog-normalized) clustered the samples by cell type, but no distinct groups were detected for PD- and control-patient derived cells (Fig. 2a). We then went on to examine differential gene expression between control- and PD-derived cell populations within every cell type and adjusted our model for differential expression for the covariate gender and for the iPSCs additionally for passage number. As ageing marks have been reported to be removed in four-factor reprogrammed cells [39], we did not include age as a covariate in the analysis of iPSCs and differentiated neurons, but only for fibroblasts.

It became evident that relevant differential gene expression between the PD- and control-group could only be observed in neuronal cells with 97 deregulated loci, but not in fibroblasts with 3 or iPSCs with 11 deregulated loci (log2FC ≥ 0.6, p-adj. < 0.1, Fig. 2b and Additional file 4: Table S2). Analysing these alterations in neuronal cells on the single gene level, genes that belong to the WNT-pathway were deregulated, i.e. *WNT3* was upregulated in PD-patient derived neurons (two-sided Mann-Whitney test, *p* < 0.05, *n* = 8 CTRL neurons and 7 PD neurons, Fig. 2c). On the pathway level, the NOS1- and CREB-pathways as well as the pathway regulating PGC1α (among others) were significantly inactivated in PD-patient derived neurons (*p* < 0.05, FDR < 0.25, n = 8 CTRL neurons and 7 PD neurons, Figs. 2d and e and Additional file 5: Table S3). Both, the PGC1α- and CREB-pathway are well-known and important regulators of neuronal cell survival [23, 67]. As such, these findings provided first implications for the usefulness of neuronal models derived from sporadic PD-patients via the iPSC stage for disease modelling and prompted us to further examine the epigenome of our cell lines across differentiation stages.

### A disease-specific piRNA/piRNA-like molecule signature is present across all differentiation stages

Next, we examined the small RNA expression patterns via NGS in all 15 fibroblast cell lines, 24 iPSC lines, two hESC lines and ten lines differentiated to midbrain neurons. When PD-patient derived cells were compared with controls, there were 26 deregulated miRNAs in fibroblasts, 34 in iPSCs and 40 in neurons (p-adj < 0.1, logFC ≥0.6, Additional file 6: Figure S3A and Additional file 7: Table S4). For mature miRNAs, the first important finding was that let-7 family members are upregulated in PD-patient derived neurons (Additional file 6: Figure S3B). The let-7 family has been reported to be deregulated in a *C. elegans* model of PD [3] and this might be another regenerative mechanism, like WNT-pathway upregulation, in PD-derived neurons.

**Fig. 3:**
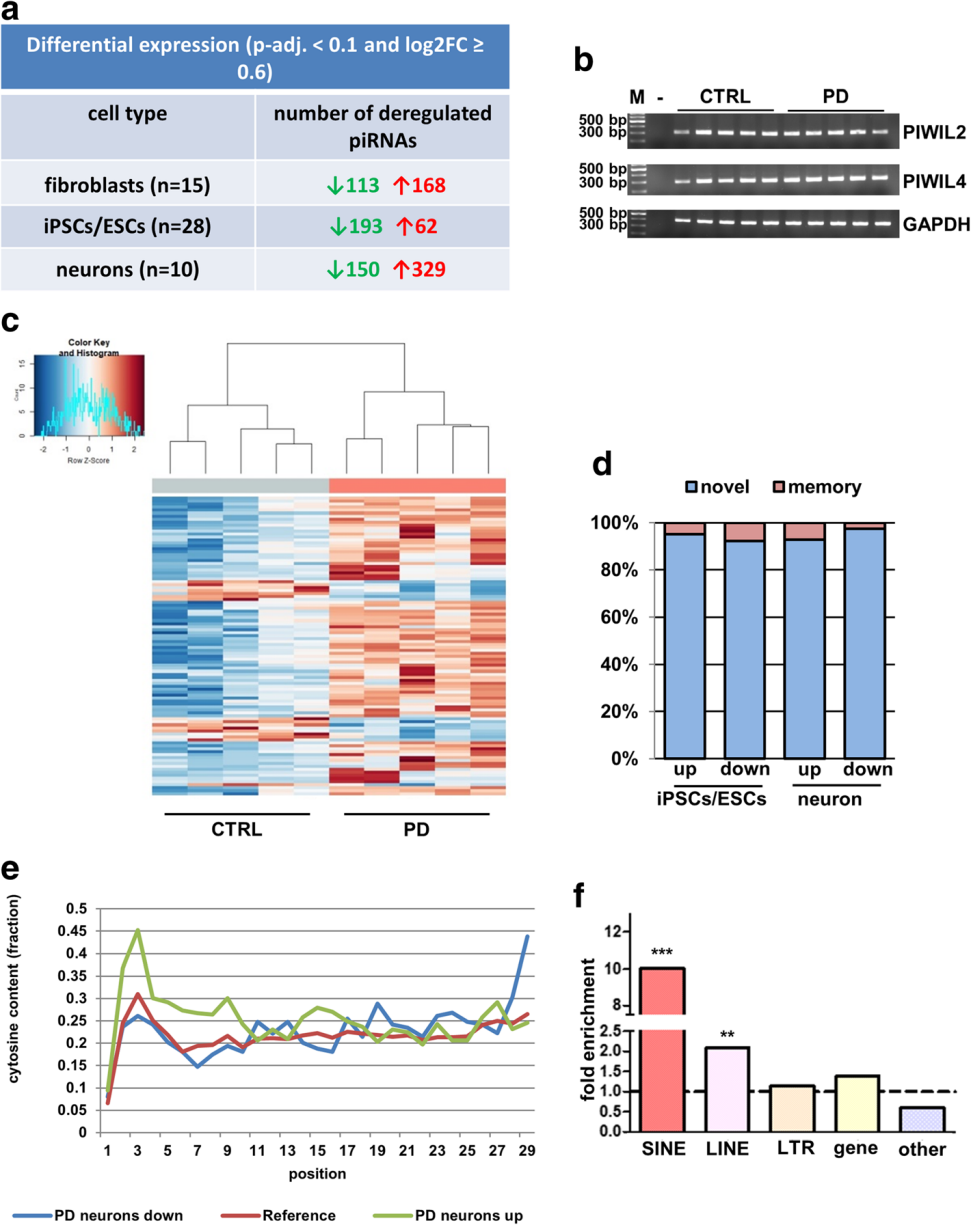
piRNAs are differentially expressed between control- and PD-patient derived cells (**a**) Differentially expressed piRNAs between control- and PD-patient derived cells (log2FC ≥ 0.6, p-adj. < 0.1) in fibroblasts, iPSCs/ESCs and neurons. **b** Semiquantitative PCR of PIWIL2 and PIWIL4 in the neurons used for the analysis of small RNA expression patterns. Both genes are expressed in cultured neurons. GAPDH was used as a loading control. A 100 bp DNA-ladder (M) and a negative control (−) were loaded together with the PCRs from control (CTRL) and PD-patient (PD) neuronal samples. **c** Heatmap and hierarchical clustering based on the TOP100 differentially expressed piRNAs in neurons (sorted by adjusted *p*-value). PD-patient derived cells (salmon) are clearly separated from control-patient derived cells (azure). **d** Memory-related piRNAs (i.e. piRNAs already differentially expressed in parental fibroblasts) are present, but constitute a minor fraction (< 10%) of all deregulated piRNAs in iPSCs/ESCs and neurons. **e** Plot of cytosine content in all deregulated piRNAs over nucleotide positions 1 to 29. In the first 10 nucleotides, cytosines are overrepresented in the upregulated piRNAs (green line) as compared to all piRNAs analysed (dark red line). This observation does not apply to the downregulated piRNAs (blue line). **f** SINE- and LINE-derived piRNAs are highly enriched in the downregulated piRNA fraction in neurons. SINE- and LINE-derived piRNAs (but not LTR- or gene-derived piRNAs) are significantly enriched in the fraction of downregulated piRNAs as compared to their abundance in the genome (two-sided chi-square test, *p* < 0.0001 and *p* < 0.01, respectively)

Even more strikingly, we found a high number of PIWI interacting RNAs (piRNAs) differentially regulated in all cell populations (Fig. 3a and Additional file 8: Table S5). We next checked if genes that control piRNA biogenesis are actually expressed in cultured neurons. Indeed, PIWIL2 and PIWIL4 expression were detectable in cultured midbrain neurons (Fig. 3b) which is similar to results that others have published from tissue in mouse [41] and human [60]. Importantly, when we examined all 15 fibroblast lines and a subset of 13 PD iPSC and 10 control iPSC lines as well as two hESC lines for total small RNA content, there were no significant differences (Kruskall-Wallis test, *p* > 0.05, Additional file 9: Figure S4A). We also asked if our library preparation included the correct length fraction of piRNAs. With our library preparation protocol, we expect the 24–32 bp piRNAs to run approximately between 150 and 160 bp, slightly higher than 22 bp mature miRNAs. The library sizes of small RNA libraries showed peaks in this size range (Additional file 9: Figure S4B). Of course, it should be noted that other RNA species, e.g. iso-mirs can also be present in these higher size ranges. As contamination of piRNAs with other RNAs, i.e. snoRNA degradation products has been a concern for IP based approaches [49], we checked the overlap between the piRNA sequences we used for piRNA mapping [66] and a snoRNA database [32]. However, as the overlap with snoRNAs was negligible and as the piRNAs identified by us showed the typical 5-prime Uridine bias, we conclude that we identified bona fide piRNA sequences. Nonetheless, as a length of 24–32 bp is another important criterion for canonical piRNAs [68] we checked the length fractions of our sequenced libraries after adapter trimming (data not shown). Due to the fact that there were less reads in the range of 24–32 bp than unique piRNA hits, we conclude that our dataset contains canonical piRNAs and piRNA-like molecules that are abundantly expressed outside of the testes as has been described by others [64]. For simplicity, piRNAs and piRNA-like molecules are referred to as piRNAs in the rest of the manuscript.

Based on the TOP100 significantly deregulated piRNAs, PD- and control midbrain neurons formed separate clusters in a hierarchical clustering analysis (Fig. 3c). However, the fraction of memory piRNAs (i.e. piRNAs that were differentially regulated between PD- and control-patient derived iPSCs/neurons and already found deregulated in fibroblasts) was rather low and always below 10% of all deregulated piRNAs (Fig. 3d). Only two piRNAs, piR-48,442 and piR-43,518, were deregulated between PD- and control-patients across all cell types (Additional file 8: Table S5).

Among the upregulated piRNAs, there was a strong enrichment of cytosine content within the bases two to nine of the piRNAs as compared to the reference of all piRNAs (Fig. 3e).

We then analysed with the annotations provided in the piRBase [66] from which elements the deregulated piRNAs were derived. Interestingly, SINE- and LINE-derived piRNAs were significantly enriched among the downregulated piRNAs as compared to the genome-wide abundance of all piRNAs analysed in our study (two-sided chi-square test, *p* < 0.0001 and *p* < 0.01, respectively, Fig. 3f). Despite massive piRNA deregulation, however, we could not find any overlap between known PD-risk loci and predicted piRNA loci in the human genome (data not shown).

We conclude that -dependent on the cell type analysed- the aberrant small RNAome is activated at different differentiation stages but only few deregulated piRNAs are shared between these stages.

### piRNA expression differences in differentiation

**Fig. 4:**
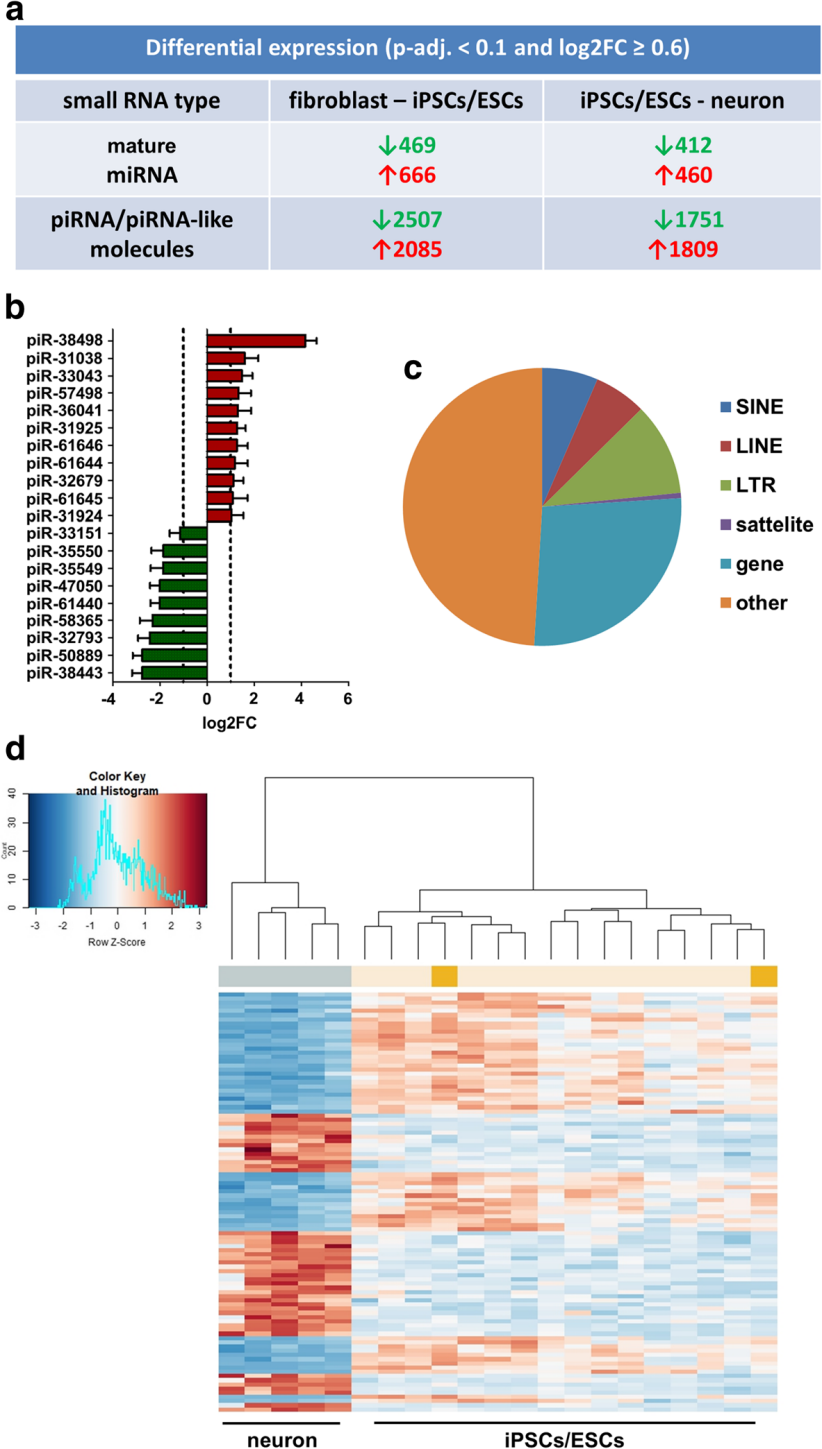
piRNAs are differentially expressed during induction of pluripotency and neuronal differentiation**. a** Analysis of differential mature miRNA and piRNA expression by DESeq2 in control-patient derived cells. During induction of pluripotency and neuronal differentiation, piRNAs are as dynamically regulated as mature miRNAs. **b** TOP20 most highly expressed (sorted by base mean as calculated by DESeq2) and differentially regulated piRNAs in the comparison iPSC/ESC vs. neuron in control-patient derived cells. **c** Pie chart of the genomic elements from which the differentially regulated piRNAs in the comparison iPSC/ESC vs. neuron are derived. SINE-derived piRNAs are significantly enriched in the fraction of deregulated piRNAs as compared to their genome-wide abundance among all piRNAs annotated by piRBase, which is set to one (two-sided chi-square test, p < 0.0001). **d** Heatmap and hierarchical clustering based on the TOP100 differentially expressed piRNAs in control iPSCs/ESCs vs. neurons (sorted by adjusted p-value). Neurons (azure) are nicely separated from iPSCs (yellow) and ESCs (gold)

As there were striking differences in piRNA expression between PD- and control-patient derived cells, we hypothesized that piRNAs should be altered by neural induction in control cells, too. Indeed, piRNAs underwent dramatic changes after induction of pluripotency and neural differentiation and were even more dynamically regulated as mature miRNAs (Fig. 4a and Additional file 10: Table S6). As many piRNAs showed a low individual abundance, we checked the 20 most abundant and differentially expressed (logFC ≥0.6 and p-adj. < 0.1) and therefore potentially most important piRNAs in the comparison iPSC vs. neuronal cells that constitute on average 19.33 ± 7.49% (mean ± SD) of all piRNA counts across neurons and iPSCs (Fig. 4b). Importantly, among all deregulated piRNAs, again SINE-derived piRNAs were overrepresented in the comparison fibroblasts (*n* = 6) vs. iPSCs/ESCs (*n* = 16) and iPSC/ESC vs. neurons (*n* = 5) (two-sided chi-square test, *p* < 0.001 and *p* < 0.0001, respectively, Fig. 4c). As expected, a hierarchical clustering based on the TOP100 differentially expressed piRNAs separated control-patient derived neurons and iPSCs (Fig. 4d).

### Tissue validation of mRNA and mi/piRNA expression patterns

**Fig. 5:**
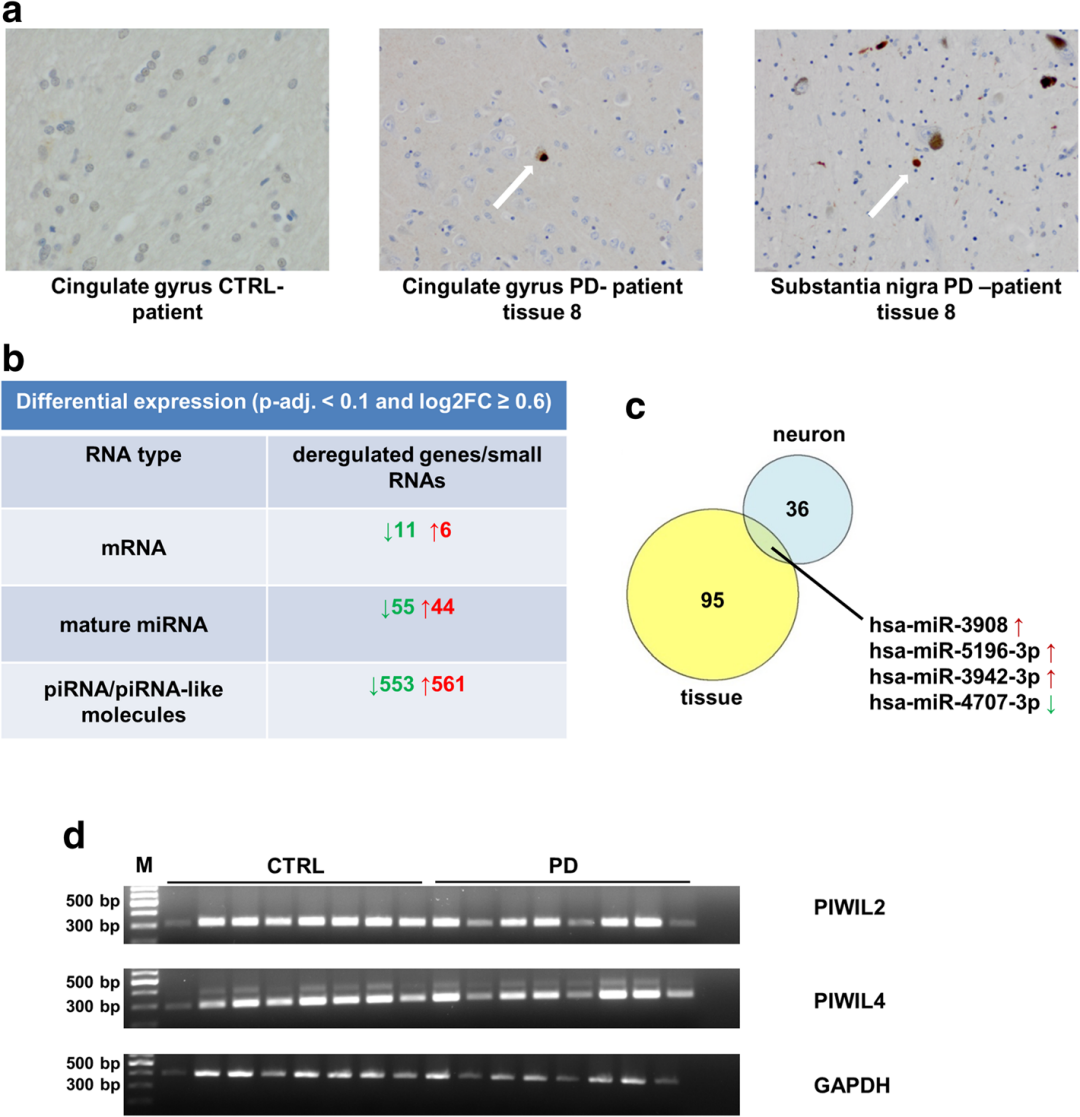
Analysis of tissue samples underscores the relevance of piRNAs in PD. **a** Histology of a cingulate gyrus section of a healthy control, a substantia nigra section from a PD-patient (PD-patient 8 from the tissue cohort) and a cingulate gyrus section of the same patient all stained with an antibody directed against aggregated α-synuclein. Lewy bodies are present both in the cingulate gyrus as well as in the substantia nigra of the PD-patient. **b** Analysis of differential mRNA, mature miRNA and piRNA expression by DESeq2. There are significant differences between control- and PD-patients in every type of RNA. **c** Venn diagram of all common upregulated and downregulated mature miRNAs (log2FC ≥ 0.6, p-adj. < 0.1) in tissues and neurons. **d** Semiquantitative PCR of PIWIL2 and PIWIL4 in the tissues used for the analysis of small RNA expression patterns. Both genes are expressed in cingulate gyrus tissue. GAPDH was used as a loading control. A 100 bp DNA-ladder (M) was loaded together with the PCRs from control (CTRL) and PD-patient (PD) cingulate gyrus samples

For the evaluation of small RNA expression pattern in PD tissue, we used material from the cingulate gyrus of eight healthy and eight Parkinson’s disease patients. We verified that the PD-patient derived tissues from the cingulate gyrus were positive for Lewy bodies (Fig. 5a). When analysing differential gene expression, we realized that oligodendrocyte marker genes were overrepresented in control patients, despite of the fact that classical cell type markers (GFAP, OLIG1/2/3, TUBB3 and RBFOX3) remained unchanged. We therefore included neuronal content (calculated as described in material and methods) as a covariate. A decreased transcriptional contribution of the oligodendrocyte lineage in Parkinson’s patients had been reported previously [22]. Importantly, the mean RPKM of the marker gene sets reported by others in mouse [6] was very specific for human cells and neuronal and glial markers were highly correlated (Additional file 11: Figure S5).

With correction for cell type abundance, there were many deregulated small RNAs at logFC ≥0.6, p-adj. *p* < 0.1 between PD- and control-patients (Fig. 5b and Additional file 12: Table S7). In our cohort, four miRNAs overlapped between tissue and neurons when control- and PD-patient derived cells were compared (Fig. 5c). As we found many deregulated piRNAs in neurons, we tested if there is expression of PIWIL2 and PIWIL4. Indeed, expression of both genes was detectable in all tissue samples (Fig. 5d).

**Fig. 6:**
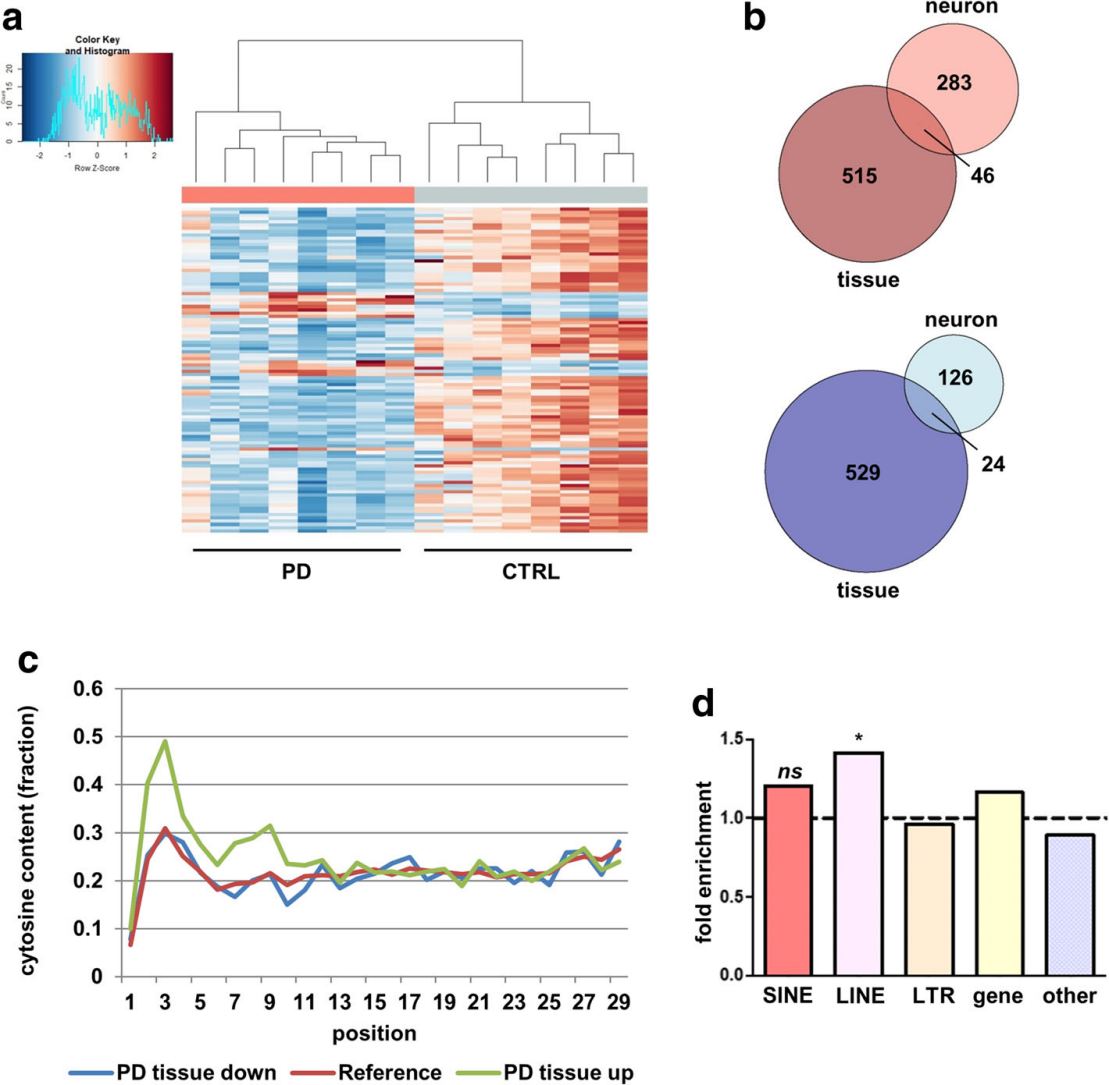
Integration with tissue data identifies disease-relevant alterations in piRNAs in neurons. **a** Heatmap and hierarchical clustering based on the TOP100 differentially expressed piRNAs in tissues (sorted by adjusted *p*-value). PD-patient tissues (salmon) are clearly separated from control-patient tissues (azure). **b** Venn diagrams of all upregulated and downregulated piRNAs in tissue and neurons. There are 70 shared piRNAs that may be suited as diagnostic markers. **c** Plot of cytosine content in all deregulated piRNAs over nucleotide positions 1 to 29 in tissue. In the first 10 nucleotides, cytosines are overrepresented in the upregulated piRNAs (green line) as compared to all piRNAs (dark red line). This phenomenon is not present in the downregulated piRNAs (blue line). **d** SINE- and LINE-derived piRNAs are enriched in the downregulated piRNA fraction in tissue. SINE- and LINE-derived piRNAs are enriched in the fraction of downregulated piRNAs as compared to their abundance in the genome, although this effect is only significant for LINE-derived piRNAs (two-sided chi-square test, *p* < 0.05)

In addition, there was abundant deregulation of piRNAs, effectively separating PD and control cases based on the TOP100 differentially expressed piRNAs (sorted by adjusted *p*-value, Fig. 6a). Although -as expected- differentially expressed piRNAs were largely different between cultured cells and tissues, there was an overlap of 70 piRNAs that deserve future evaluation as diagnostic marker (Fig. 6b). We again observed an overrepresentation of cytosines among the upregulated piRNAs in the second to ninth base (Fig. 6c). Importantly, both SINE- and LINE-derived piRNAs were enriched among the downregulated piRNAs in tissue, as was described for neurons above, although this reached significance only for LINE-derived elements (two-sided chi-square test, *p* < 0.05, Fig. 6d).

Of note, enrichment of cytosines within the first 10 bp and overrepresentation of SINE- and LINE-derived elements among the downregulated piRNAs was not consistently retained in fibroblasts and iPSCs. Therefore, aberrant piRNA expression is unmasked in differentiated neurons, but there is no significant epigenetic memory present within the small RNA fraction.

### Only cell type specific differences exist in CpG methylation

We then examined differential methylation between control and PD cell lines. We successfully sequenced more than 400,000 CpGs at a coverage ≥5× measured in every sample. We analysed 15 fibroblast lines (*n* = 6 in the CTRL group and *n* = 9 in the PD group), 28 pluripotent stem cell preparations (*n* = 16 in the CTRL group and *n* = 12 in the PD group), ten midbrain dopaminergic neurons (*n* = 5 in the CTRL group and n = 5 in the PD group). Sequences were highly enriched in CpG islands, CpG island shores and functional elements, as expected. There were differences in global methylation patterns between cell types, but not between PD- and control-patient derived cells (Additional file 13: Figure S6 A-C). Even after restricting the analysis to autosomes and stringent removal of low variability regions there were only very few differentially methylated CpGs (cut-off Δmeth ≥0.2, p-adj. < 0.1, data not shown) in fibroblasts (36 CpGs), iPSCs (6 CpGs) or neurons (45 CpGs) and no difference in the mean methylation pattern of any well-covered (at least 5 CpGs per promoter with 5× coverage) gene promoter of known monogenic PD genes (Additional file 13: Figure S6D).

Thus, global methylation patterns that had been found to be reduced in late stage disease [33], were unaltered in vitro as judged by RRBS. This finding was confirmed in the tissues where no differences in methyl-cytosine staining could be observed between PD- and control-patients (Additional file 14: Figure S7). In comparison with a previous study [12] that reported global methylation differences between PD- and control-patient derived neurons we used another protocol for iPSC derivation as well as neuronal differentiation. We therefore conclude that in our experimental setup there is no methylation-based epigenetic memory or any disease-specific alteration in the CpG context in sporadic PD-patient derived cells.

Finally, we analysed mtDNA methylation patterns on the basis of our RRBS data as well as mtDNA mass and mtDNA deletions by real-time PCR. There were no significant differences between PD- and control-patient derived cells but again only between cell types themselves (Kruskall-Wallis test followed by Dunn’s multiple comparison test, Additional file 15: Figure S8).

## Discussion

Systematic screening for phenotypes in cells established from well-defined cohorts of sporadic PD-patients has not been performed, yet. Therefore, as part of the ForIPS consortium, we aimed to elucidate if sporadic PD-patient derived cells carry any alterations as compared to matched control patients. We can show that PD-patient derived cells show a specific small RNA signature in every cell type examined.

Several studies have suggested similarities between cells established from genetic and sporadic cases in certain assays [12, 18, 54]. To identify the molecular basis of such potential disease phenotypes we performed a comprehensive analysis of mRNA and small RNA expression patterns as well as methylation analysis at single base resolution in a unique cohort of fibroblasts, iPSCs and differentiated midbrain neurons from sporadic PD-patients. In classical phenotypic assays, the midbrain neurons differentiated from sporadic PD-patients did not show a cellular phenotype [59].

Nonetheless, on the mRNA level, the pathway regulating PGC1α (peroxisome proliferator-activated receptor-γ coactivator-1α) is inactivated in PD-patient derived midbrain neurons, which was previously reported to be involved in disease-specific phenotypes in an A53T model of PD [53] and is a hallmark of PD pathology [67]. PGC1α is a master regulator of mitochondrial function and protects neurons from apoptotic cell death under stress conditions in in vitro models of PD [53, 67]. In addition, the CREB-(cAMP response element binding protein) pathway, which is a known neuroprotective pathway [23], was impaired in PD-patient derived neurons. CREB proteins, which are transcription factors mediating cAMP responses, (besides their function in cell survival) are involved in numerous processes in the nervous system, e.g. memory formation and neurogenesis [44]. Importantly, CREB-activity is modulated by PGC1α [9] connecting the identified pathways on a functional level. On a single gene level we found a significant PD-associated effect on the transcriptome in differentiated neurons, while fibroblasts or iPSCs showed no differences. Among the genes deregulated in neuronal cells, there were several WNT-pathway members. These have been reported to be hypermethylated in PD [65] and upregulation of these genes in our midbrain neurons might serve as a protective mechanism as reported in a PD mouse model [31]. In addition, genes and pathways regulating neurodevelopment that function downstream of WNT signals and play a role in midbrain development, i.e. *LMX1B* [29] and *OTX2* [12], have been suggested to be involved in PD pathogenesis. We add the WNT-pathway members WNT3, ANT3A and WNT9B to this catalogue.

We furthermore show that neuronally differentiated cells show striking similarities with diseased tissue on the level of small RNAs. Here we detected the differential regulation of many PIWI interacting RNAs and/or piRNA-like molecules. Importantly, it has been reported by others that the orthologues of PIWI-proteins are expressed in the mammalian brain [37, 41]. Although piRNAs were first described in testes where they show the highest abundance [2], a large number of subsequent studies have identified their presence in the mammalian brain including studies on human tissue [10, 30, 41, 48, 52]. PiRNAs play an important role for retrotransposon silencing in the brain [41] and retrotransposon activation contributes to the genetic mosaicism in neurons in *Drosophila* [46]. Retrotransposon encoding loci are hypomethylated in mice deficient for a mouse orthologue of PIWI (MILI) [41]. On the functional level, chromatin modification and transcriptional repression are guided by piRNAs [19]. Moreover, piRNAs modulate synaptic plasticity in *Aplysia* neurons via CREB2 in response to serotonin [49] as well as dendritic spine size in mammalian cells [30]. Of note, others have recently described deregulated piRNA expression in Alzheimer’s disease, emphasizing the relevance of piRNAs for neurodegenerative diseases [48, 52]. We identfied a number of piRNAs that are shared between diseased PD-patient brain tissue and cultured neuronal cells. Importantly, the overrepresentation of LINE- and SINE-derived piRNAs among the downregulated piRNAs points towards a failure of PD-patient derived neurons to properly silence these elements. In addition, by analysing the size fraction of the piRNAs, we can show that both bona fide piRNAs as well as piRNA-like molecules contribute to the pool of deregulated sequences. This is important, as the catalogues of deregulated piRNAs in brain tissue from other diseases [10, 52, 56] will most likely also contain a significant number of piRNA-like sequences. These are abundant outside of the testes [64], but their function is less well understood than that of canonical piRNAs.

It has been reported that sporadic PD-patient derived neurons show aberrant protein turnover, altered morphology and methylation patterns when compared to control-patient derived cells [12, 18, 54]. We extend these findings by completely tracing the cells on epigenomic and transcriptomic level from the primary fibroblasts through the iPSC stage to the neuronally differentiated stage. More importantly, we can show that the alterations are detectable with the widely used protocols of OKSM reprogramming followed by differentiation to midbrain neurons. Interestingly, neither on the methylation nor the small RNA level there were changes that would comprise a significant epigenetic memory. Only the PD-patient derived neurons had alterations that were comparable to the diseased tissue. However, we cannot completely rule out the possibility that epigenetic memory persists in epigenetic modifications that were not analysed in our study, e.g. on histones, as has been reported in other systems [17].

There may be some other limitations to our study that should be shortly mentioned: 1) We used a classical retroviral four factor reprogramming cocktail. Reprogramming with non-integrating vectors like Sendai-viruses or pure RNA may provide a platform for functional studies less prone to effects due to reprogramming factor integration into the genome. 2) Our study is focused on the analysis of mRNA/small RNA expression and DNA methylation patterns. Biochemical validation of the effects of deregulated piRNAs/piRNA-like molecules and pathways will be an active area of future research when the appropriate tools are available. 3) Further examination of cortically differentiated neurons and/or laser capture microdissection of purified dopaminergic neurons from the substantia nigra could support and refine the conclusions on disease etiology of different types of neurons both in the in vitro and in vivo setting.

Nonetheless, our study implies that disease modelling with cells derived from sporadic PD cases is possible. We conclude that although four factor reprogramming removes ageing associated marks [39] and iPSCs from healthy individuals are equivalent to ESCs [5, 8], Parkinson’s disease derived neuronal cells show disease-specific alterations on transcriptomic and epigenetic level. More importantly, we show that both in neurons and in post-mortem brain tissue from PD-patients, piRNAs are deregulated in a similar fashion, which is a striking new finding both in the field of PD as well as cell biology research. We support this findings with a total number of more than 200 next-generation sequencing runs (DNA methylation, microRNAs, mRNAs) on one of the largest collectives of high-quality PD-derived cell preparations that is available in the field.

## Additional files

Additional file 1: **Table S1.** Cohort statistics with details about age, gender and all other covariates used for differential expression models. (XLSX 19 kb)

Additional file 2: **Figure S1.** Analysis of pluripotency marker expression. (TIF 403 kb)

Additional file 3: **Figure S2.** mRNA based analysis of neuronal differentiation. (TIF 1295 kb)

Additional file 4: **Table S2.** Differential expression analysis for mRNAs in fibroblasts, iPSCs/ESCs and neurons for the comparison PD vs. CTRL. (XLSX 18407 kb)

Additional file 5: **Table S3.** Gene set enrichment analysis in neurons for the comparison PD vs. CTRL (based on mRNA-RPKM data) for Biocarta and KEGG pathways. (XLSX 1121 kb)

Additional file 6: **Figure S3.** Differential expression of mature miRNAs in vitro. (TIF 447 kb)

Additional file 7: **Table S4.** Differential expression analysis for mature miRNAs in fibroblasts, iPSCs/ESCs and neurons for the comparison PD vs. CTRL. (XLSX 718 kb)

Additional file 8: **Table S5.** Differential expression analysis for piRNAs/piRNA-like molecules in fibroblasts, iPSCs/ESCs and neurons for the comparison PD vs. CTRL. (XLSX 10389 kb)

Additional file 9: **Figure S4.** Small RNA content analysis and library size distribution. (TIF 491 kb)

Additional file 10: **Table S6.** Differential expression analysis for piRNAs/piRNA-like molecues and mature miRNAs for the comparison control fibroblasts vs. control iPSCs/ESCs and control iPSCs/ESCs vs. control neurons. (XLSX 7706 kb)

Additional file 11: **Figure S5.** Analysis of cell type abundance and marker genes in tissues. (TIF 524 kb)

Additional file 12: **Table S7.** Differential expression analysis for mRNAs, mature miRNAs and piRNAs/piRNA-like molecules in tissues for the comparison PD vs. CTRL. (XLSX 9950 kb)

Additional file 13: **Figure S6.** Global statistics on RRBS and analysis of differential methylation. (TIF 351 kb)

Additional file 14: **Figure S7.** Immunohistochemical staining for methyl-cytosine in all eight control- and PD-patients. (TIF 3846 kb)

Additional file 15: **Figure S8.** Analysis of mtDNA parameters. (TIF 416 kb)
