# Supplementary figures and images for: Sporadic Parkinson’s disease derived neuronal cells show disease-specific mRNA and small RNA signatures with abundant deregulation of piRNAs
Source: Acta Neuropathol Commun. 2018 Jul 10;6:58. doi: 10.1186/s40478-018-0561-x (PMC6038190; doi:10.1186/s40478-018-0561-x)

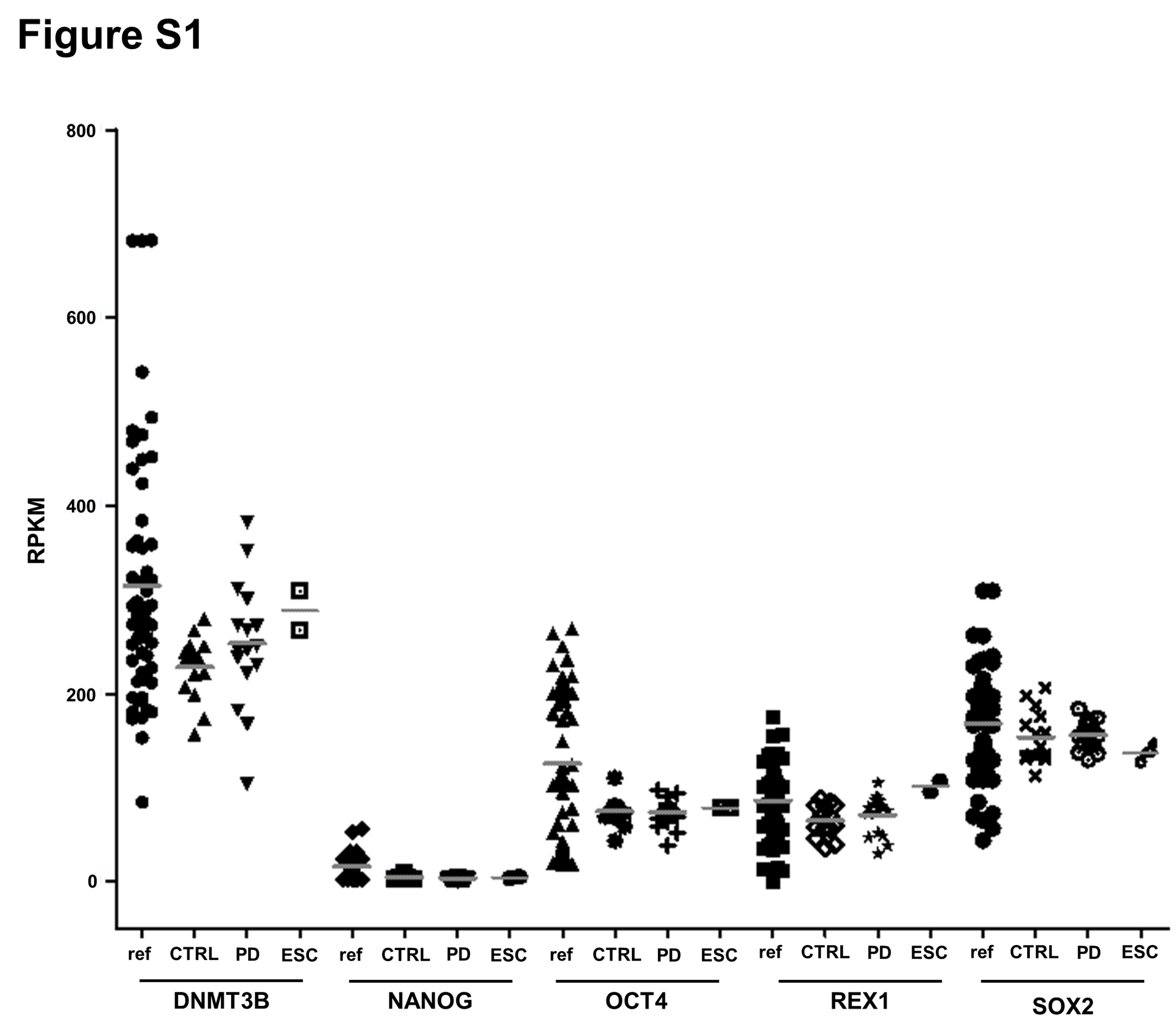

Supplement: Supplementary file 2 — Figure S1. Analysis of pluripotency marker expression. (TIF 403 kb) [file 40478_2018_561_MOESM2_ESM.tif]

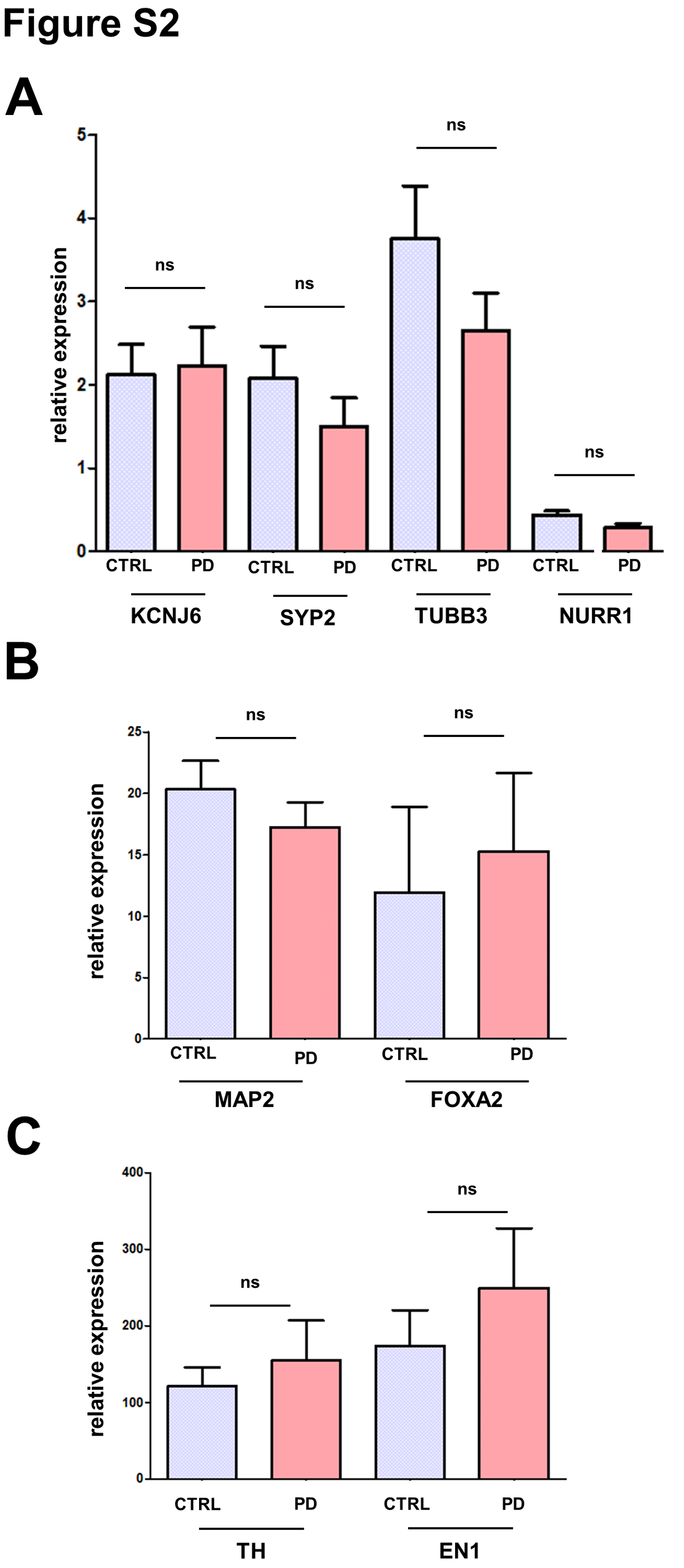

Supplement: Supplementary file 3 — Figure S2. mRNA based analysis of neuronal differentiation. (TIF 1295 kb) [file 40478_2018_561_MOESM3_ESM.tif]

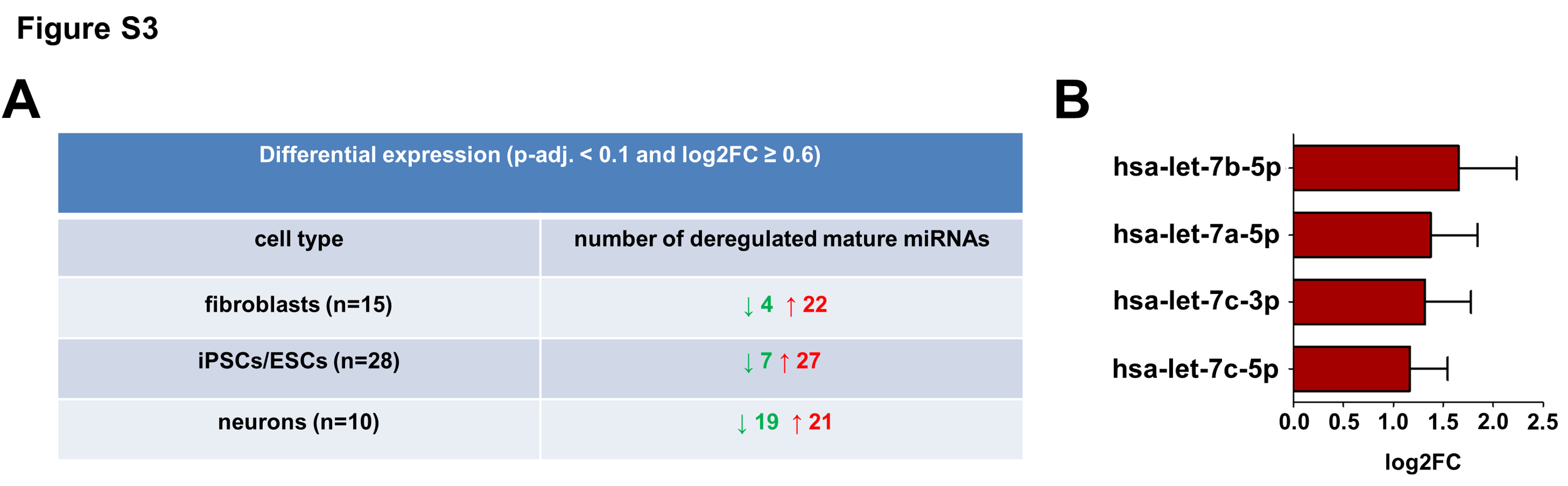

Supplement: Supplementary file 6 — Figure S3. Differential expression of mature miRNAs in vitro. (TIF 447 kb) [file 40478_2018_561_MOESM6_ESM.tif]

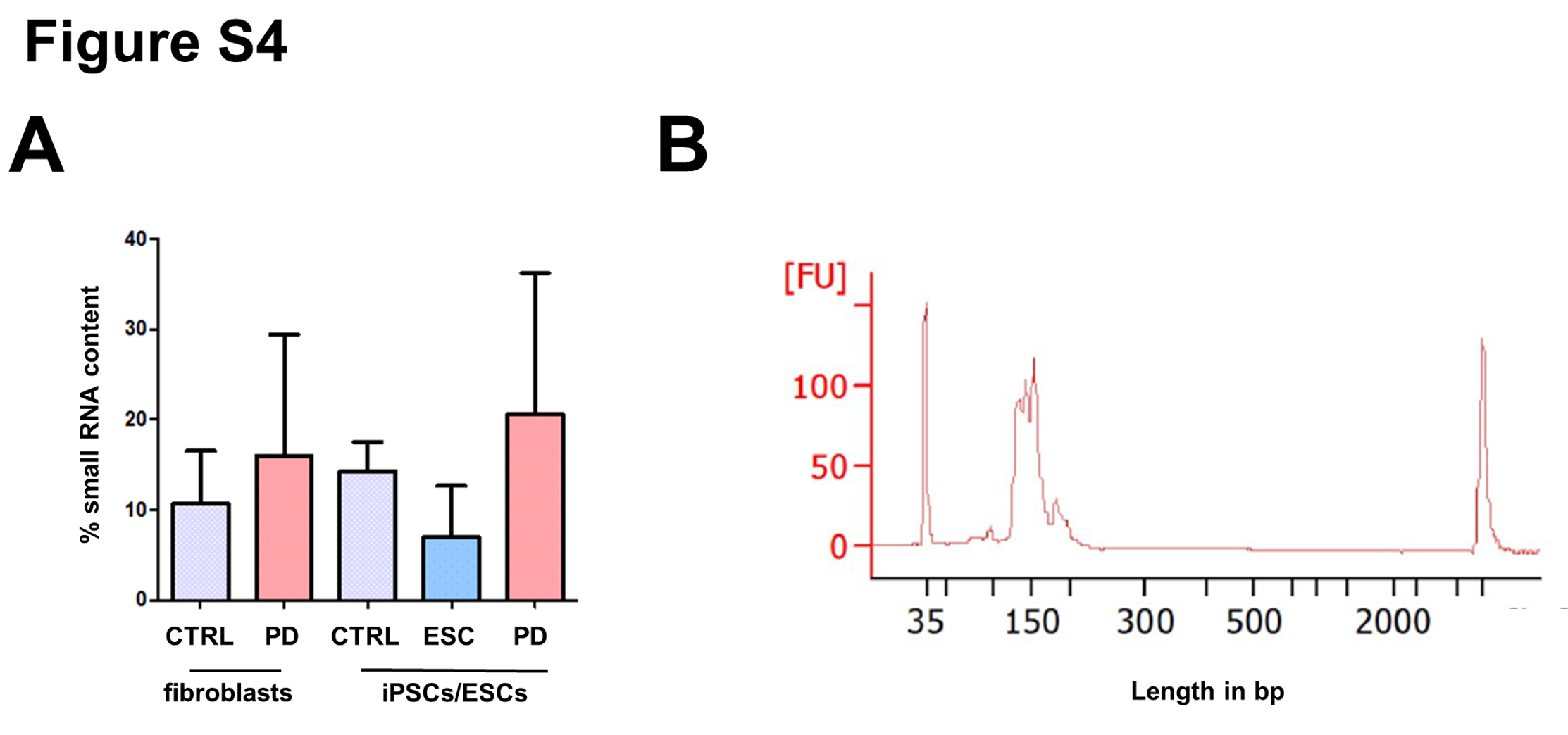

Supplement: Supplementary file 9 — Figure S4. Small RNA content analysis and library size distribution. (TIF 491 kb) [file 40478_2018_561_MOESM9_ESM.tif]

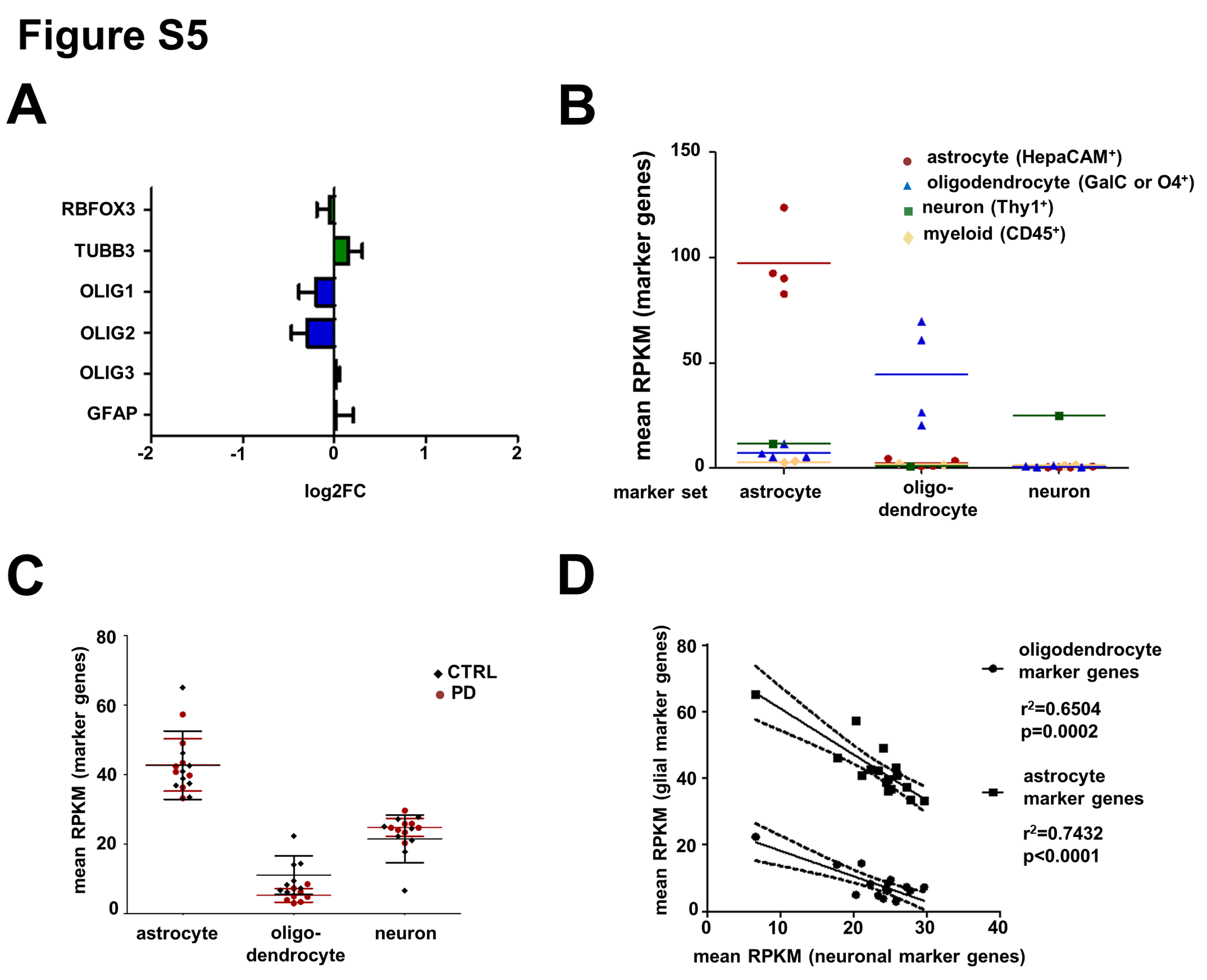

Supplement: Supplementary file 11 — Figure S5. Analysis of cell type abundance and marker genes in tissues. (TIF 524 kb) [file 40478_2018_561_MOESM11_ESM.tif]

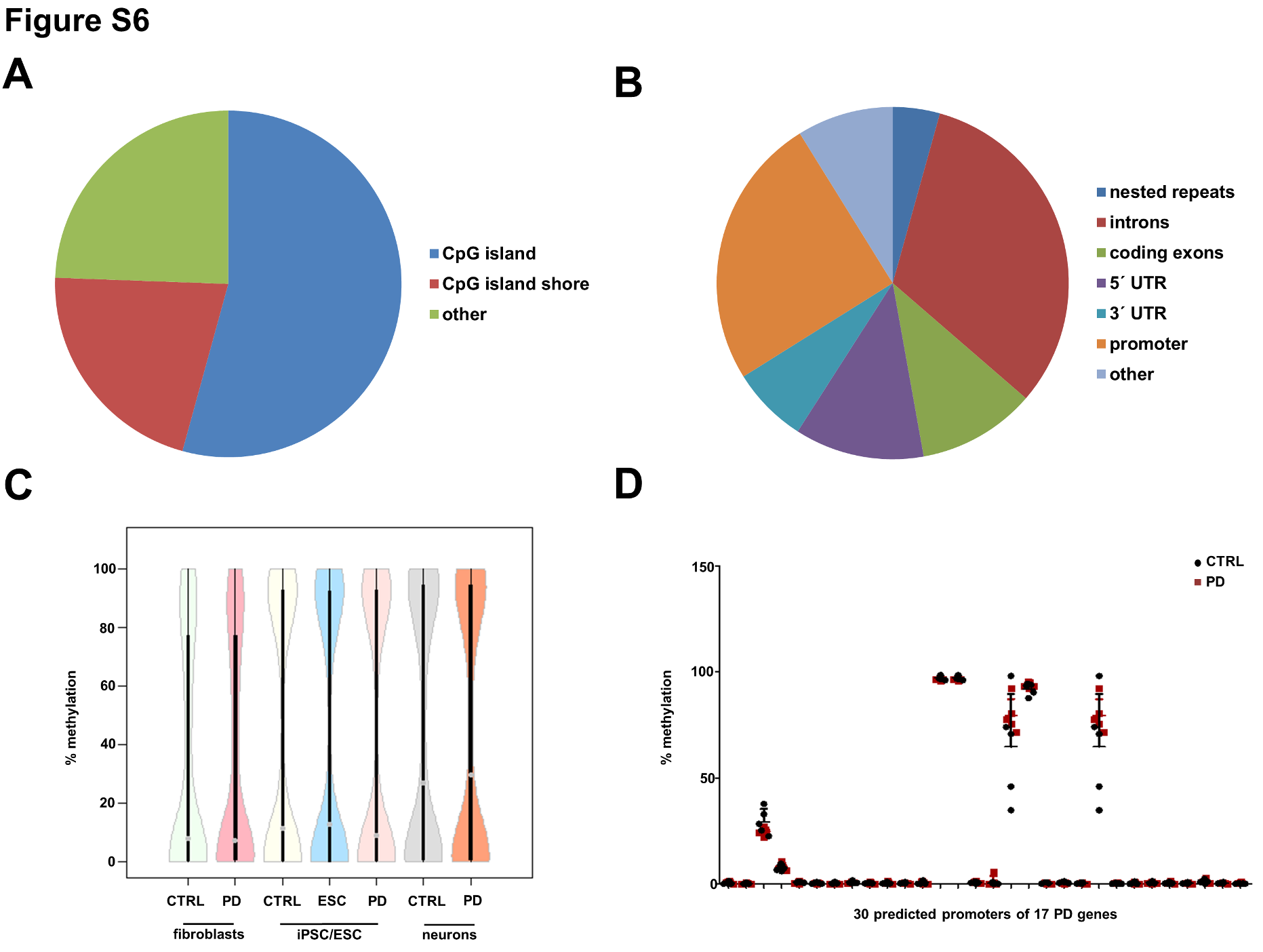

Supplement: Supplementary file 13 — Figure S6. Global statistics on RRBS and analysis of differential methylation. (TIF 351 kb) [file 40478_2018_561_MOESM13_ESM.tif]

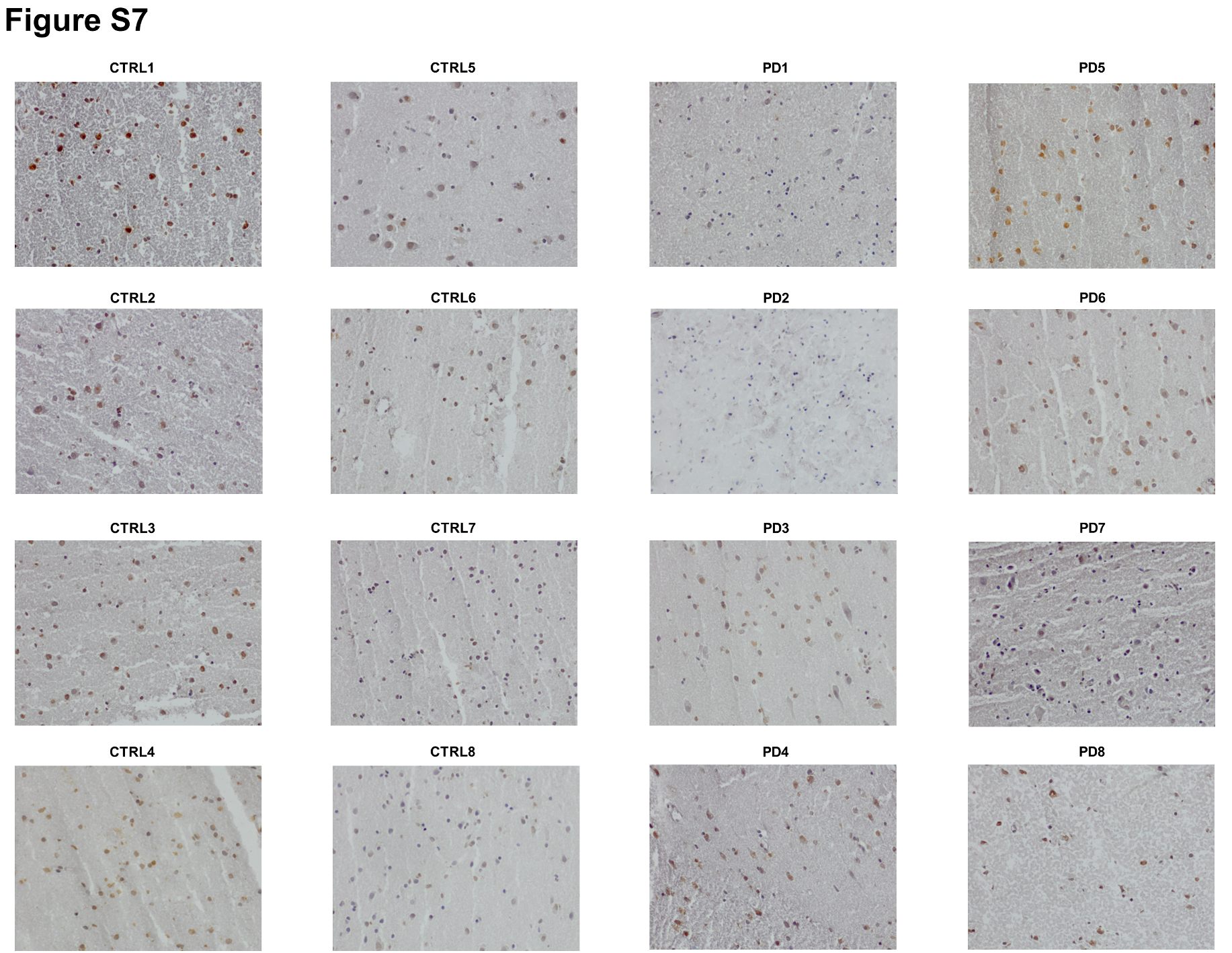

Supplement: Supplementary file 14 — Figure S7. Immunohistochemical staining for methyl-cytosine in all eight control- and PD-patients. (TIF 3846 kb) [file 40478_2018_561_MOESM14_ESM.tif]

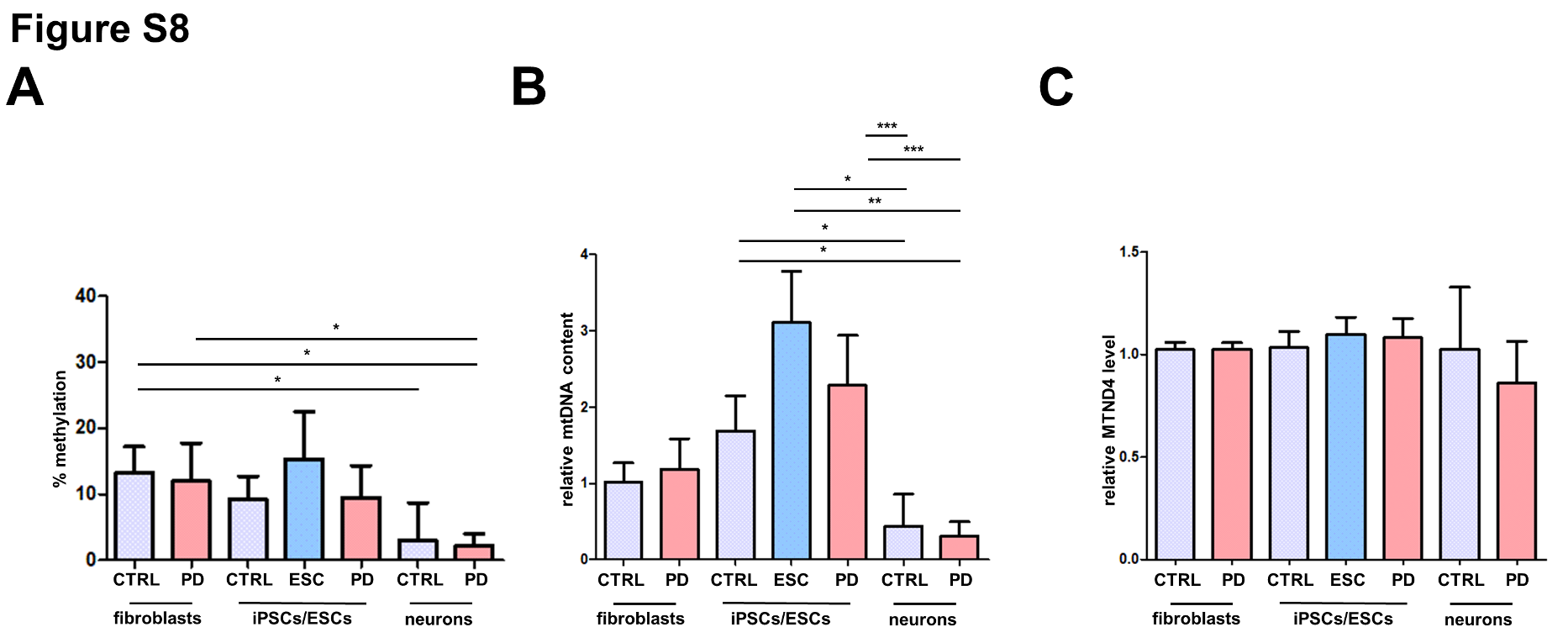

Supplement: Supplementary file 15 — Figure S8. Analysis of mtDNA parameters. (TIF 416 kb) [file 40478_2018_561_MOESM15_ESM.tif]
